# Maternal Cytokines CXCL12, VEGFA, and WNT5A Promote Porcine Oocyte Maturation via MAPK Activation and Canonical WNT Inhibition

**DOI:** 10.3389/fcell.2020.00578

**Published:** 2020-07-07

**Authors:** Xin Liu, Yuchen Hao, Zhekun Li, Jilong Zhou, Hongmei Zhu, Guowei Bu, Zhiting Liu, Xudong Hou, Xia Zhang, Yi-Liang Miao

**Affiliations:** ^1^Institute of Stem Cell and Regenerative Biology, College of Animal Science and Veterinary Medicine, Huazhong Agricultural University, Wuhan, China; ^2^Key Laboratory of Agricultural Animal Genetics, Breeding and Reproduction (Huazhong Agricultural University), Ministry of Education, Wuhan, China; ^3^College of Animal Science and Veterinary Medicine, Huazhong Agricultural University, Wuhan, China; ^4^College of Life Science and Technology, Huazhong Agricultural University, Wuhan, China; ^5^The Cooperative Innovation Center for Sustainable Pig Production, Huazhong Agricultural University, Wuhan, China

**Keywords:** porcine oocyte, CXCL12, VEGFA, WNT5A, *in vitro* maturation, embryo development

## Abstract

Maternal regulatory factors endow the oocyte with developmental competence *in vivo*, which might be absent in current *in vitro* maturation (IVM) systems, thereby compromising oocyte quality. In the present study, by employing RNA sequencing data analysis, we expect to identify potential contributing factors to support porcine oocyte maturation through binding to their receptors on the oolemma. Here, C-X-C motif chemokine ligand 12 (CXCL12), vascular endothelial growth factor A (VEGFA), and Wingless-type MMTV integration site family member 5A (WNT5A), termed CVW, are selected and confirmed to be important maternal cytokines for porcine oocyte maturation. Combined supplementation of CVW promotes the nuclear maturation percentage from 57.2% in controls to 75.9%. More importantly, these maternal cytokines improve the developmental potential of matured oocytes by parthenogenesis, fertilization, and cloning, as their blastocyst formation efficiencies and total cell numbers are increased. CVW supplementation also enlarges perivitelline space and promotes cumulus expansion, which results in a more complete transzonal projection retraction on the zona pellucida, and a reduced incidence of polyspermy in fertilized oocytes. Meanwhile, inhibiting the CVW receptor-mediated signaling pathways severely impairs oocyte meiotic resumption and cumulus expansion during IVM. We further determine that maturation improvement by CVW is achieved through activating the MAPK pathway in advance and inhibiting the canonical WNT pathway at the end of the IVM period. These findings provide a new combination of three cytokines to promote the porcine IVM process, which also holds potential to be used in human assisted reproduction technologies as well as in other species.

## Introduction

*In vitro* maturation (IVM) is an important reproductive technique to obtain viable oocytes for pig embryo and piglet production, which has been widely used to create genetically modified pigs for breed improvement or human disease research. For example, by inserting the mouse *UCP1* gene, pigs have been created with reduced fat deposition and improved thermogenic capacity (Zheng et al., 2017). Another pig model with mutant huntingtin expression successfully exhibits features of Huntington’s disease for further clinical study (Yan et al., 2018). Additionally, pigs are considered to be the best candidate for human organ generation via xenotransplantation, and their endogenous retrovirus has been genetically inactivated to eliminate safety concerns for future applications (Niu et al., 2017). So far, all these studies require a large number of porcine embryos derived by *in vitro* fertilization (IVF) or somatic cell nuclear transfer (SCNT) techniques. However, these embryos often display unresolved developmental defects, such as high rates of embryonic losses during the preimplantation stage, and the extremely low efficiency in producing offspring (Romar et al., 2016; Yuan et al., 2017; Ruan et al., 2018). For reasons not fully clear, oocytes obtained after IVM are less competent than their *in vivo*-produced counterparts, which is believed to be responsible for the poor developmental phenotype of IVF or SCNT embryos, thereby limiting the practical use of these techniques in agriculture and biomedicine.

In general, the maternal environment provides regulatory factors to ensure the growth of follicles and oocytes in an optimized manner for pregnancy. However, the current IVM culture system cannot entirely recapitulate the maternal environment for oocyte maturation, especially for the oocytes harvested from immature gilts. Thus, many studies have focused on the selection and characterization of cytokines, which are enriched in ovarian follicular fluid or secreted by granulosa cells and the reproductive system, to promote oocyte development during IVM. Among the most studied cytokines are epidermal growth factor (EGF) (Lonergan et al., 1996; Prochazka et al., 2000; Valleh et al., 2017), insulin-like growth factor 1 (IGF1) (Kiapekou et al., 2005; Pereira et al., 2012; Oberlender et al., 2013), leukemia inhibitory factor (LIF) (Dang-Nguyen et al., 2014; Mo et al., 2014; An et al., 2018), and vascular endothelial growth factor (VEGF) (Luo et al., 2002; Yan et al., 2012; Kere et al., 2014). Recently, combined supplementation of fibroblast growth factor 2 (FGF2), LIF and IGF1 (termed FLI) was proved to improve porcine oocyte competence in an indirect way, probably by activating the mitogen-activated protein kinase (MAPK) signaling pathway in cumulus cells (Yuan et al., 2017). By contrast, we asked if there are other maternal factors that could directly bind to their receptors on the plasma membrane of oocytes, modulate downstream signaling pathways, and contribute to meiotic resumption and subsequent preimplantation development *in vitro*.

Here, by performing high-throughput RNA sequencing (RNA-seq) analysis, three intercellular signaling receptor genes, C-X-C motif chemokine receptor 4 (*CXCR4*), fms-like tyrosine kinase 1 (*FLT1*), and frizzled class receptor 5 (*FZD5*), were highly expressed in porcine *in vivo*-matured oocytes and fertilized embryos. Meanwhile, the mRNAs of their corresponding ligands (termed CVW), C-X-C motif chemokine ligand 12 (CXCL12), VEGFA, and Wingless-type MMTV integration site family member 5A (WNT5A), were abundant in reproductive tissues. Therefore, we assumed that CXCL12-CXCR4, VEGFA-FLT1, and WNT5A-FZD5 interactions may play a crucial role in porcine oocyte development. The present study was aimed at assessing the effect of combined CVW supplementation on oocyte maturation in our pig IVM medium, and dissect the signaling pathways modulated by this modified medium.

## Materials and Methods

Reagents and chemicals used in this study were purchased from Sigma-Aldrich (St. Louis, MO, United States) unless otherwise indicated.

### Experimental Design

Experiment 1: Light on the IVM of cumulus oocyte complexes (COCs). Different concentrations of CVW and their receptor inhibitors were individually added to IVM medium for COC culture. The first polar body (PB1) extrusion percentage was counted at 42 h of maturation. Moreover, combined CVW supplementation was performed to determine and assess the PB1 extrusion percentage, maturation morphology, chromosome and spindle morphology, cortical granule (CG) distribution, early apoptosis signal, transzonal projection (TZP) retraction, polyspermy, cumulus expansion, and signaling pathways. Partial experiments were treated with combined receptor inhibitors as another experimental group. The detailed method of each experiment is shown below.

Experiment 2: Light on the IVM of denuded oocytes. Different concentrations of CVW were individually added to IVM medium for denuded oocyte culture, and the PB1 extrusion percentages were counted at 42 h of maturation. Moreover, combined treatments of CVW and their receptor inhibitors were also carried out to determine the PB1 extrusion percentage.

Experiment 3: Light on the *in vitro* culture (IVC) of parthenogenetically activated (PA) oocytes. Different concentrations of CVW and their receptor inhibitors were individually added to IVC medium, termed porcine zygote medium 3 (PZM3), for PA embryo culture. The PA blastocyst percentage was counted after culture for 7 days *in vitro*.

Experiment 4: Light on the IVC of CVW-matured oocytes. PA, IVF and SCNT were performed by using oocytes matured with or without CVW supplementation. The blastocyst percentage of each group was counted after culture for 7 days *in vitro*. Moreover, the blastocyst cell number, inner cell mass (ICM) cell number, apoptotic cell number were determined through immunostaining. The detailed method of each experiment is shown below.

### Oocyte Collection and IVM

Oocyte collection and IVM were performed as previously described with some modifications (Lai and Prather, 2003; Wang et al., 2017). Briefly, porcine ovaries were obtained from a local abattoir and transported to the laboratory at 35–38°C within 2–4 h. Follicular fluid from 3 to 6 mm antral follicles was aspirated with an 18-gauge needle and syringe. Then, COCs with several layers of cumulus cells and even oocyte cytoplasm were selected from follicular fluid and washed in IVM medium (TCM-199 medium supplemented with 10% porcine follicular fluid, 5 μg/mL insulin, 10 ng/mL EGF, 0.6 mM cysteine, 0.2 mM pyruvate, 25 μg/mL kanamycin and 5 IU/mL of each equine and human chorionic gonadotrophin). Approximately 30 COCs per well were placed into a 96-well plate containing 150 μL IVM medium and cultured for 42 h under 5% CO_2_ in air at 38.5°C. After maturation, cumulus cells were removed by gentle pipetting in 0.1% (w/v in PBS) hyaluronidase, and oocytes with PB1 extrusion were regarded as matured oocytes. For denuded oocyte IVM, COCs were firstly pipetted in 0.1% hyaluronidase to remove cumulus cells before transfer into IVM medium. The maturation morphologies of oocytes were defined as previously described (Yuan et al., 2017): Type I, incomplete PB1 extrusion with a narrow perivitelline space; Type II, complete PB1 extrusion with a narrow perivitelline space; Type III, complete PB1 extrusion with a wide perivitelline space.

### Cytokine and Inhibitor Treatment

According to the manufacturer’s instructions, recombinant human CXCL12 (300–28A, PeproTech, Rocky Hill, NJ, United States) and VEGFA (100-20, PeproTech) were diluted in 0.1% (w/v in PBS) bovine serum albumin (BSA); recombinant mouse Wnt5a (GF146, Sigma) was directly packaged. CVW were then individually added to IVM medium or IVC medium with a final concentration of 0, 1, 5, 10, 25, 50, and 100 ng/mL. AMD3100 (S8030, Selleck Chemicals, Houston, TX, United States), Axitinib (S1005, Selleck Chemicals) and Box5 (681673, Sigma) were receptor inhibitors for CXCR4, FLT1 and FZD5, respectively. AMD3100 and Box5 were dissolved in water, while Axitinib was dissolved in dimethyl sulfoxide. According to previous studies (Jenei et al., 2009; Kere et al., 2014; Zhang et al., 2018), AMD3100 (0, 0.05, 0.1, 0.5, and 1 μM), Axitinib (0, 0.1, 1, 5, and 10 μM), and Box5 (0, 10, 50, 100, and 500 μM) were individually added to IVM and PZM3 media. For the FLI test, 40 ng/mL FGF2 (RD-233-FB, R&D Systems, Minneapolis, MN, United States), 20 ng/mL LIF (LIF1010, Merck Millipore, Billerica, MA, United States), 20 ng/mL IGF1 (100-11R3, PeproTech) were added as previously described (Yuan et al., 2017) to our IVM medium. All cytokines and inhibitors were packaged into single-use aliquots and stored at −80°C.

### Parthenogenetic Activation

Parthenogenetic activation was performed as previously described with some modifications (Lai and Prather, 2003; Kong et al., 2014). Briefly, oocytes were placed in an activation chamber with electrodes 1 mm apart containing activating medium. Activation was induced with 2 direct current pulses of 1.2 kV/cm for 30 μs on a BTX Electro-Cell Manipulator (BTX ECM2100, San Diego, CA, United States). The activated oocytes were then washed and transferred to PZM3 medium, and cultured under 5% CO_2_ in air at 38.5°C.

### IVF and Polyspermy Assessment

*In vitro* fertilization was performed as previously described with some modifications (Kong et al., 2014). Briefly, frozen pig semen (Begenda, Beijing, China) was thawed in 50°C for 16 s and washed in pre-heated 10 mL modified Tris-buffered medium (mTBM) for 5 min. After 1,900 × *g* centrifugation for 5 min and resuspension, a 50-μL sperm suspension (5 × 10^5^ sperms/mL in mTBM) was added to a drop of 50 μL mTBM medium containing 30 matured oocytes. After fertilization for 4–6 h under 5% CO_2_ in air at 38.5°C, redundant sperm on the zona pellucida was removed by gentle pipetting in mTBM medium, and the fertilized oocytes were then cultured in PZM3 medium as described for PA.

For polyspermy assessment, putative zygotes were collected for pronuclei counting at 18 h after IVF. Briefly, zygotes were washed in 0.2% polyvinyl alcohol (w/v in PBS) and fixed in 4% paraformaldehyde (w/v in PBS) for 30 min at room temperature. After another three washes, DNA was stained with 15 μg/mL 4,6-diamidino-2-phenylindole (DAPI, Beyotime, Shanghai, China) for 3 min at room temperature. Zygotes were finally mounted on glass slides with a drop of anti-fade mounting medium (Beyotime), and captured by using a confocal microscope and a standard objective (Zeiss LSM 800, Oberkochen, Germany) with the same scanning settings.

### SCNT

Somatic cell nuclear transfer was performed as previously described with some modifications (Lai and Prather, 2003). Briefly, pig fetal fibroblast cells (2–5 passages) were cultured in Dulbecco’s modified Eagle’s medium (Thermo Fisher Scientific, Waltham, MA, United States) containing 0.5% fetal bovine serum (HyClone, South Logan, UT, United States) for 72 h, and digested by trypsin-EDTA solution to use as donor cells. Matured oocytes were placed into manipulation medium containing 7.5 μg/mL cytochalasin B for 5 min. Enucleation was performed with a 20-μm glass pipette by aspirating PB1 and adjacent cytoplasm to remove the nucleus from the oocyte, and a single donor cell was injected into the perivitelline space. Because oocyte activation could be achieved during electrical fusion, these reconstructed embryos were then activated and cultured as described for PA.

### Chromosome and Spindle Staining

Oocytes cultured for 42 h were fixed in 4% paraformaldehyde for 30 min, permeabilized in 0.5% Triton X-100 (v/v in PBS) for 30 min, blocked in 5% BSA (w/v in PBS) for 2 h, and incubated with anti-α-Tubulin-FITC antibody (1:800 dilution, F2168, Sigma) for 1 h to visualize the spindle morphology (Nie et al., 2017). After three washes and DNA staining with DAPI, oocytes were mounted and captured by using a confocal microscope and an oil objective with the same scanning settings. All steps were performed at room temperature. Normal morphologies of chromosomes and spindles were classified as previously described (Nie et al., 2017): Type I, chromosomes are aligned on the midline with spindle-shaped microtubules alongside; Type II, punctate chromosomes are detected in cobweb-shaped microtubules at the metaphase plate. Disorganized spindles containing condensed chromatin were regarded as abnormal Type III, and multipolar spindles with misaligned chromosomes were regarded as abnormal Type IV.

### Cortical Granule Distribution Assessment

Oocytes cultured for 42 h were fixed, permeabilized, and blocked as mentioned above. Then, oocytes were incubated with FITC-conjugated peanut lectin (1:200 dilution, L7381, Sigma) for 1 h at room temperature to visualize CGs (Zhang et al., 2018). After three washes, oocytes were mounted and observed by using a confocal microscope and a standard objective. Fluorescent signals at the equatorial plane, defined as the section in which the oocyte displays the greatest diameter, were then captured with the same scanning settings. The classification of CG distribution was based on previous studies (Liu et al., 2018a; Zhang et al., 2018): Type I, a monolayer CG distribution under the oolemma; Type II, a diffused CG distribution in the ooplasm; Type III, no CG distribution in matured oocytes.

### Early Apoptosis Detection

Oocytes cultured for 42 h were collected for examining apoptosis signals by using Annexin V-FITC Apoptosis Detection Kit (C1062S, Beyotime) according to the manufacturer’s instructions (Wang et al., 2017). Subsequently, oocytes were immediately fixed in 4% paraformaldehyde for 30 min at room temperature. After three washes, apoptotic fluorescent signals at the oocyte’s equatorial plane were captured as described for CG distribution assessment. The classification of apoptosis signals was based on a previous study (Wang et al., 2017): Type I, no apoptosis signal on the oolemma; Type II, a few signals on the oolemma; Type III, a strong monolayer signal on the oolemma.

### Transzonal Projection Detection

Cumulus oocyte complexes cultured for 0, 22, and 42 h were collected for labeling actin filaments to visualize TZPs (Yuan et al., 2017). Briefly, COCs were fixed, permeabilized, and blocked as mentioned above. Then, COCs were incubated with Actin-Tracker Green (1:50 dilution, C1033, Beyotime) for 1 h at room temperature. After three washes and DNA staining with DAPI, the fluorescent signals of TZPs at the oocyte’s equatorial plane were captured as described for CG distribution assessment. The number of TZPs was counted by using Image-Pro PLUS software (Media Cybernetics, Rockville, MD, United States).

### Cumulus Expansion Assessment

Cumulus oocyte complexes cultured for 42 h were collected and captured by using bright-field microscopy (Nikon Ti-E, Tokyo, Japan). The diameters of COCs were evaluated by using Image-Pro PLUS software. COCs were characterized as non-expanded and fully expanded, when their diameters were less than 300 μm or larger than 600 μm, respectively. When the diameters of COCs were between 300 and 600 μm, these were regarded as partially expanded COCs.

### Quantitative PCR Analysis

Cumulus cells were stripped from 30 COCs cultured for 0, 22, and 42 h by using 0.1% hyaluronidase. Total RNA was isolated by RNAprep pure Micro Kit (DP420, TIANGEN, Beijing, China), followed by DNA removal and reverse transcription PCR by HiScript II Q RT SuperMix plus gDNA wiper (R223-01, Vazyme, Nanjing, China). Samples were then quantified by ChamQ SYBR Quantitative PCR (qPCR) Master Mix (Q321-02, Vazyme) on CFX96 Real-Time PCR Detection System (Bio-Rad, Hercules, CA, United States). The results from 0 h samples were set as 1, and were normalized to the internal control gene *GAPDH*. All primer sequences are listed in Supplementary Table S1. Data are shown as the fold change = 2^–ΔΔCt^ mean ± standard deviation (SD).

### Blastocyst Cell Number Count

PA, IVF and SCNT blastocysts cultured for 7 days were collected for cell number counting. Briefly, blastocysts were fixed, permeabilized, and blocked as mentioned above. Then, samples were incubated with SRY-box transcription factor 2 (SOX2) antibody (1:200 dilution, sc-365823, Santa Cruz Biotechnology, Santa Cruz, CA, United States) for 24 h at 4°C to visualize ICM cells. After three washes, samples were treated with DyLight 488 Goat Anti-Mouse IgG (1:500 dilution, A23210, Abbkine, Wuhan, China) for 1 h at room temperature. After three washes and DNA staining with DAPI, blastocysts were mounted and captured by using fluorescence microscopy (Nikon Ti-E) with the same exposure settings. Total cell numbers were counted as the number of nuclei stained blue. ICM cell numbers were counted as the number of SOX2-positive nuclei stained green (Bou et al., 2017).

### TUNEL Assay

PA, IVF and SCNT blastocysts cultured for 7 days were collected for terminal deoxynucleotidyl transferase dUTP nick end labeling (TUNEL) to visualize apoptotic cells. Briefly, blastocysts were fixed and permeabilized as mentioned above. One Step TUNEL Apoptosis Assay Kit (C1089, Beyotime) was then used according to the manufacturer’s instructions. After three washes and DNA staining with DAPI, blastocysts were mounted and captured by using fluorescence microscopy (Nikon Ti-E) with the same exposure settings. Apoptotic cell numbers were counted as the number of TUNEL-positive nuclei stained red. Apoptosis rates were calculated as the ratio of apoptotic cell numbers to total cell numbers (Liu et al., 2018b).

### MAPK and WNT Signaling Pathway Analysis

Oocytes cultured for 0, 22, and 42 h were collected for assessing signaling pathways. For immunostaining, oocytes were fixed, permeabilized, and blocked as mentioned above. Then, samples were incubated with primary antibodies against p44/42 MAPK (1:200 dilution, #4695, CST, Danvers, MA, United States), phospho-p44/42 MAPK (1:200 dilution, #4370, CST) or β-catenin (1:100 dilution, #8480, CST) for 24 h at 4°C. After three washes, samples were treated with DyLight 549 Goat Anti-Rabbit IgG (1:500 dilution, A23320, Abbkine) for 1 h at room temperature. After another three washes and DNA staining with DAPI, the fluorescent signal of each pathway at the oocyte’s equatorial plane was captured as described for CG distribution assessment. Fluorescence intensity was finally determined by using ImageJ software (NIH, Bethesda, MD, United States). Briefly, images were converted into 8-bit format, and the region of interest (ROI) was defined as the size of oocytes by auto threshold adjustment with default setting. The average fluorescence intensity per unit area within ROI was defined as mean gray value in Set Measurements window.

For immunoblotting, 200 oocytes in each group were lysed in radioimmunoprecipitation assay buffer containing a cocktail of protease and phosphatase inhibitors (P1050, Beyotime). Denatured samples were separated on 10% acrylamide gels and transferred to polyvinylidene fluoride membranes (Merck Millipore) for 1 h at 100 V. Membranes were then blocked in Tris-buffered Saline Tween-20 (TBST) buffer containing 10% non-fat milk (w/v) for 4 h and incubated with phospho-MAPK (1:1000 dilution) or β-catenin (1:1000 dilution) antibodies overnight at 4°C. After three washes in TBST, membranes were incubated with HRP-labeled Goat Anti-Rabbit IgG (1:1000 dilution, A0208, Beyotime) for 2 h. The immunoblots were visualized by using Pierce ECL Western Blotting Substrate (Thermo Fisher Scientific) on ImageQuant LAS 4000 imaging system (GE Healthcare, Chicago, IL, United States). Finally, the same membranes were immersed in stripping buffer (Beyotime), blocked in 10% non-fat milk, and reprobed with MAPK antibody (1:1000 dilution) to detect its total amount. All steps were performed at room temperature unless stated otherwise.

### RNA-Seq Data Analysis

Publicly available datasets were analyzed in this study (Kim et al., 2018; Kong et al., 2020). These data can be found in the Gene Expression Omnibus database^1^ under accession number GSE139512 and GSE108570. For GSE139512, fragments per kilobase of exon per million mapped fragments (FPKM) values of all genes in oocytes and embryos were calculated by Cufflinks tool. For GSE108570, read counts of all genes in reproductive tissues were calculated by featurecount tool. Both RNA-seq data were mapped on the reference sus scrofa genome 10.2. Next, based on the KEGG database^2^ and MeSH browser^3^, 517 genes which encode intercellular signaling ligands and receptors were summarized, and their expression values were extracted from two gene lists mentioned above (Supplementary Table S2). Receptor genes with average FPKM values > 10 were defined as highly expressed receptors in oocytes and embryos, and the read counts of their corresponding ligand genes in different reproductive tissues were ranked in descending magnitude. The 5 most highly expressed ligands were chosen for the subsequent study.

### Statistical Analysis

Protein sequences were aligned by using DNAMAN software (Lynnon Biosoft, San Ramon, CA, United States). All experiments were repeated at least three times. Data are represented as the mean ± SD and analyzed using Student’s *t-*test or one-way Analysis of Variance (ANOVA) with Duncan multiple range test with SPSS Statistics 20 software (IBM Corporation, Armonk, NY, United States). Values of *P* < 0.05 are considered significant. Differences are shown with ^∗^, ^∗∗^, ^∗∗∗^, or different letters a, b, c.

## Results

### Selection of Potential Cytokines From Transcriptomes of Pig Oocytes, Embryos, and Reproductive Tissues

**FIGURE 1:**
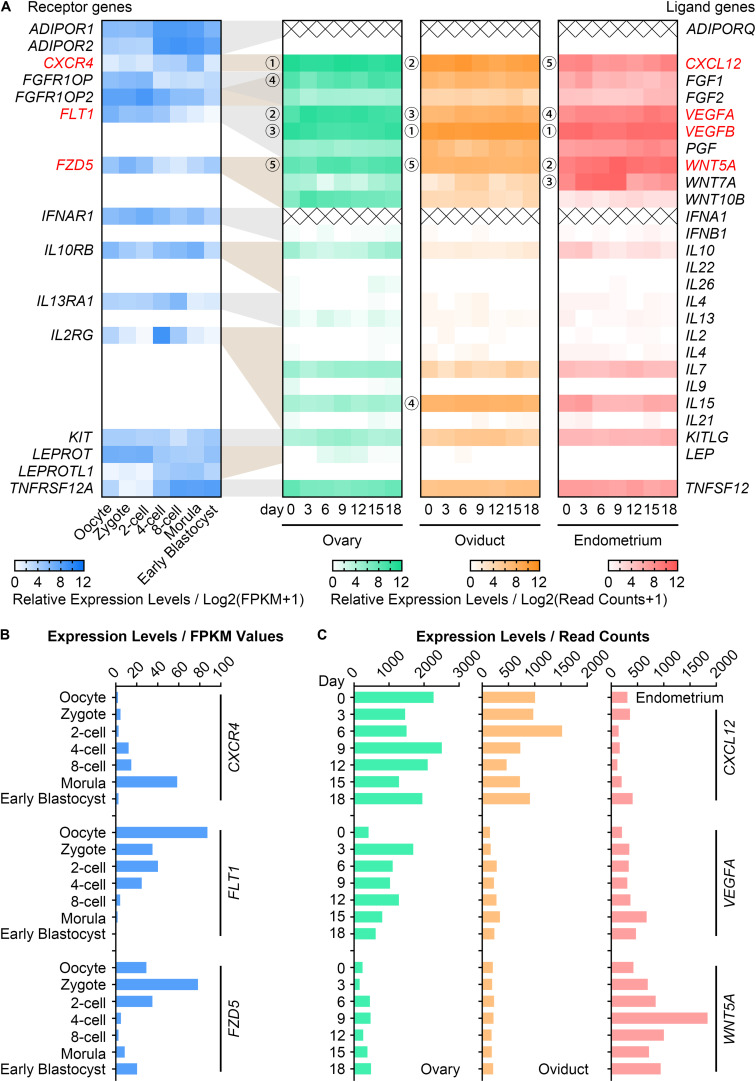
Gene expression of CVW cytokines and their receptors in porcine oocytes, embryos and reproductive tissues. **(A)** Heatmap illustrating the relative expression patterns of 15 candidate receptor genes (blue, average FPKM > 10) in porcine *in vivo*-derived oocytes and fertilized embryos at different stages, as well as their corresponding ligand genes in porcine ovary (green), oviduct (orange), and endometrium (pink) during the oestrous cycle. Cross symbols stand for not available in the present data. Circled numbers represent the top five genes with the highest expression levels in each tissue. **(B)** Expression levels of CVW corresponding receptor genes, *CXCR4*, *FLT1*, *FZD5*, in *in vivo*-derived oocytes and fertilized embryos at different stages. **(C)** Expression levels of *CXCL12*, *VEGFA*, *WNT5A* in porcine ovary, oviduct and endometrium during the oestrous cycle.

To ascertain the potential cytokines that may participate in maternal-embryo interactions, we summarized 517 genes which encode intercellular signaling ligands and receptors from the KEGG database and MeSH browser (Supplementary Table S2). The expression pattern of each gene in the RNA-seq data of pig *in vivo*-derived oocytes, fertilized embryos (Kong et al., 2020) and female reproductive system (Kim et al., 2018) were then collected (Supplementary Table S2). Among these, we found 15 highly expressed (average FPKM value > 10) receptor genes in oocytes and embryos (Figure 1A). For their corresponding ligand genes, the 5 most highly expressed genes in descending magnitude were *CXCL12*, *VEGFA*, *VEGFB*, *FGF1*, *WNT5A* in the ovary; *VEGFB*, *CXCL12*, *VEGFA*, *IL15*, *WNT5A* in the oviduct; *VEGFB*, *WNT5A*, *WNT7A*, *VEGFA*, *CXCL12* in the endometrium (Figure 1A). We thus speculated that at least CXCL12-CXCR4, VEGFA/VEGFB-FLT1, and WNT5A-FZD5 interactions may be essential for porcine oocyte and embryo development. Considering that VEGFA has been widely investigated on oocyte maturation in different species (Luo et al., 2002; Yan et al., 2012; Kere et al., 2014), we used VEGFA, not VEGFB, in our study. Figures 1B,C showed the FPKM values of *CXCR4*, *FLT1*, *FZD5* in oocytes and embryos, and the dynamic changes of *CXCL12*, *VEGFA*, *WNT5A* in reproductive tissues during the oestrous cycle. We noticed that the *CXCR4*, *FLT1*, *FZD5* transcripts peaked in morulae, matured oocytes, and zygotes, respectively (Figure 1B). In addition, *CXCL12* was enriched in the ovary and oviduct, while *VEGFA* and *WNT5A* were individually plentiful in the ovary and endometrium (Figure 1C).

Due to the absence of CXCL12, VEGFA, WNT5A in our pig oocyte and embryo culture formula, we hypothesized that combined supplementation of CVW in the medium could provide a more similar maternal environment, and thus promote both oocyte maturation and embryo development *in vitro*. Initially, we aligned recombinant CVW amino acid sequences with porcine proteins. The sequence homologies of CVW receptor-binding domains were 96.6, 96.4, and 100.0%, respectively (Supplementary Figure S1), suggesting that these recombinant human and mouse proteins are suitable for pig.

### Effects of Individual Cytokines on Oocyte Maturation and Preimplantation Development

**FIGURE 2:**
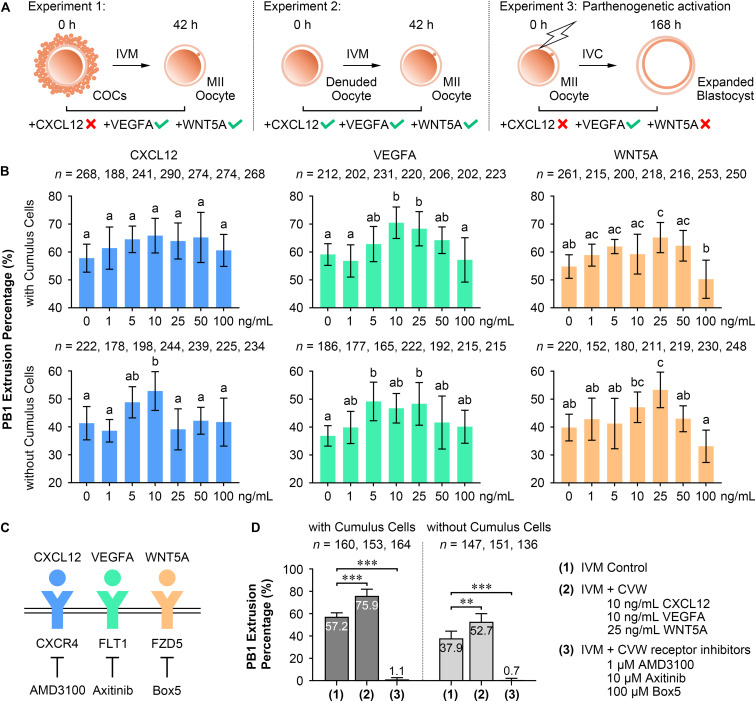
Effects of CVW and their receptor inhibitors on porcine oocyte maturation and embryo development. **(A)** Experimental design to determine the optimal concentrations of CVW for oocyte maturation and embryo development. Tick symbols indicate that it has beneficial effects on *in vitro* maturation (IVM) or *in vitro* culture (IVC). Cross symbols indicate that it has no significant effect on IVM or IVC. **(B)** Individual effects of CVW on first polar body (PB1) extrusion of oocytes cultured with or without cumulus cells. Error bars represent the SD in four to five replicates. Different superscripts indicate *P* < 0.05 (one-way ANOVA with Duncan’s test). **(C)** Proposed interactions between CVW and their receptors on the oolemma, and inhibitions on CVW receptors by using AMD3100, Axitinib, and Box5, respectively. **(D)** Combined effects of CVW and their receptor inhibitors on PB1 extrusion of oocytes cultured with or without cumulus cells. Error bars represent the SD in five replicates. ^∗∗^*P* < 0.01, ^∗∗∗^*P* < 0.001 (Student’s *t*-test).

To test our hypothesis, CVW were individually added to oocyte IVM medium and embryo IVC medium, termed PZM3 medium. PB1 extrusion and PA blastocyst formation were investigated to determine the optimal effects. For oocytes cultured with intact cumulus cells (Experiment 1, Figures 2A,B), supplementation of VEGFA (10 and 25 ng/mL) and WNT5A (25 ng/mL), but not CXCL12, enhanced the efficiency of producing metaphase II (MII) oocytes. We further examined the individual functions of CVW on denuded oocyte maturation (Experiment 2, Figures 2A,B). The PB1 extrusion was apparently improved in maturation medium supplemented with 10 ng/mL CXCL12, 5 and 25 ng/mL VEGFA, and 25 ng/mL WNT5A. Next, efficient CVW concentrations in PZM3 medium were evaluated (Experiment 3, Figure 2A and Supplementary Figure S2A). Surprisingly, no significant developmental differences were observed in CXCL12- and WNT5A-treated groups at concentrations ranging from 1 to 100 ng/mL. The percentage of PA embryos reaching the blastocyst stage was only increased in groups cultured with 25–50 ng/mL VEGFA, which is partially in accordance with a recent study (Kere et al., 2014).

Although individual CVW supplementation displayed different results on meiotic resumption and preimplantation development, we asked whether inhibition of CVW receptor-mediated signals would affect these two processes. Receptor inhibitor AMD3100 (Zhang et al., 2018), Axitinib (Kere et al., 2014), and Box5 (Jenei et al., 2009) were then utilized to specifically block the signaling pathways initiated by CXCR4, FLT1 and FZD5, respectively (Figure 2C). The results showed a decreased percentage of PB1 extrusion when different doses of AMD3100 (0.1, 0.5, and 1 μM), Axitinib (1, 5, and 10 μM) and Box5 (10, 50, 100, and 500 μM) were added to the maturation medium (Supplementary Figure S2B). Intriguingly, these three inhibitors also impaired PA blastocyst formation in a dose-dependent manner (Supplementary Figure S2B). Together, these findings indicate that CVW receptor-mediated signaling pathways are involved in the IVM and IVC process even without CVW supplementation in IVM and PZM3 media. One explanation is that some unknown factors in follicular fluid or secreted by embryos could activate these pathways during oocyte and embryo development.

### Combined Supplementation of CVW Promotes Oocyte Nuclear and Cytoplasmic Maturation

In the following study, we focused on the beneficial effects of CVW on porcine oocyte maturation. CVW (CXCL12, 10 ng/mL; VEGFA, 10 ng/mL; WNT5A, 25 ng/mL), as well as the CVW receptor inhibitors (AMD3100, 1 μM; Axitinib, 10 μM; Box5, 100 μM), were then added in combination to IVM medium at their optimal concentrations. As anticipated, CVW could enhance the PB1 extrusion percentage when COCs (CVW, 75.9%; Control 57.2%) or denuded oocytes (CVW, 52.7%; Control 37.9%) were cultured in maturation medium (Figure 2D), and the improvement achieved by combined treatment on COCs (CVW, 18.8%) was obviously higher compared to the individual treatment (VEGFA, 11.4%; WNT5A, 10.4%) (Supplementary Figure S2C). Meanwhile, combined supplementation with receptor inhibitors almost completely inhibited the maturation process (Figure 2D). Notably, CVW not only increased the PB1 extrusion percentage, it also improved the PB1 extrusion morphology, as the percentage of MII oocytes with complete PB1 extrusion and an expanded perivitelline space was elevated (Type III: CVW, 54.1%; Control 23.9%) (Supplementary Figure S2D).

**FIGURE 3:**
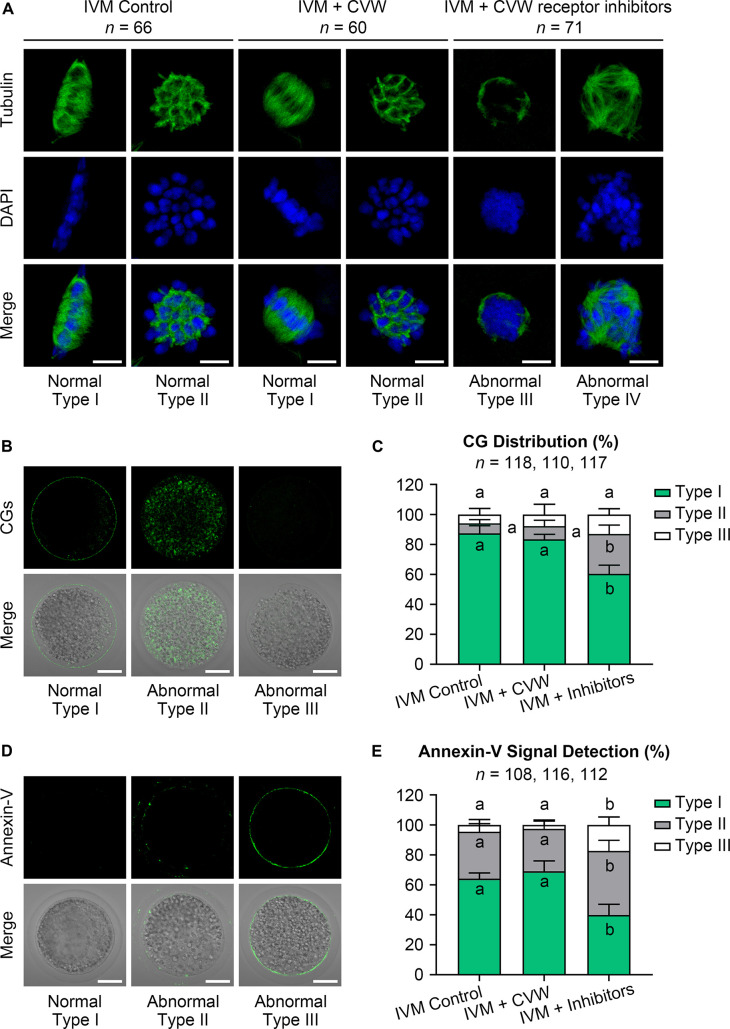
Effects of CVW and their receptor inhibitors on the quality of IVM oocytes. **(A)** Representative images of normal (Type I, II) and abnormal (Type III, IV) spindle morphologies (green) and chromosome alignments (blue) in control, CVW-treated, and inhibitor-treated oocytes. Scale bar = 5 μm. **(B)** Representative images of normal (Type I) and abnormal (Type II, III) cortical granule (CG) distributions (green) in oocytes after IVM. Scale bar = 30 μm. **(C)** Percentage of oocytes with different CG distributions in control, CVW-treated, and inhibitor-treated groups. Error bars represent the SD in four replicates. Different superscripts indicate *P* < 0.05 (one-way ANOVA with Duncan’s test). **(D)** Representative images of normal (Type I) and abnormal (Type II, III) annexin-V signal (green) types in oocytes after IVM. Scale bar = 30 μm. **(E)** Percentage of oocytes with different annexin-V signal types in control, CVW-treated, and inhibitor-treated groups. Error bars represent the SD in four replicates. Different superscripts indicate *P* < 0.05 (one-way ANOVA with Duncan’s test).

Given that oocyte development comprises nuclear and cytoplasmic maturation, both were investigated in oocytes cultured with CVW or their receptor inhibitors. For nuclear and cytoplasmic maturation, we respectively examined the chromosome and spindle morphologies by immunostaining. Both CVW-matured and control oocytes displayed spindle-shaped microtubule formations with well-ordered chromosomes at the metaphase plate (Type I, Figure 3A), or cobweb-shaped structures containing chromosomes (Type II, Figure 3A). There were no significant differences of these morphologies between CVW-matured and control oocytes. In the inhibitor-treated group, almost all oocytes showed unorganized spindles with chromatin condensation (Type III, Figure 3A), or multipolar spindles with misaligned chromosomes (Type IV, Figure 3A). The distribution of CGs was then detected as another cytoplasmic maturation indicator. More than 80% of MII oocytes (Figure 3C) showed monolayer CGs beneath the oolemma (Type I, Figure 3B) in control and CVW-treated groups. By contrast, the percentage of oocytes with this typical CG distribution declined to nearly 60% when cultured with inhibitors (Figure 3C). Moreover, we assessed early apoptosis occurrence in oocytes via annexin-V staining. The results demonstrated that CVW receptor inhibition could apparently enrich (∼60%) the apoptotic signal on the oolemma (Type II and III, Figures 3D,E), while the percentage of positive-stained oocytes was only 30–35% in control and CVW-treated groups (Figure 3E).

### Supplementation of CVW in IVM Medium Accelerates Transzonal Projection Retraction to Prevent Polyspermy

**FIGURE 4:**
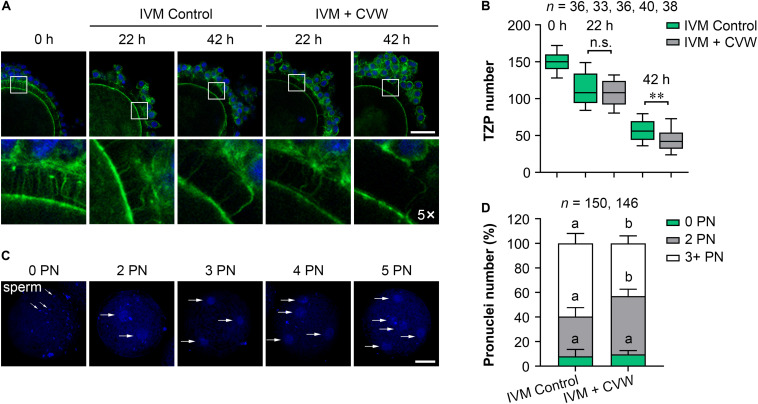
Effects of CVW on transzonal projection (TZP) retraction during IVM and polyspermic penetration after fertilization. **(A)** Representative images of actin-labeled TZPs (green) in control and CVW-treated cumulus oocyte complexes (COCs) at 0, 22, 42 h during IVM. Cumulus cell nuclei are stained blue. The hollow squares represent five-fold enlarged images of TZPs in different groups. Scale bar = 30 μm. **(B)** Box plot shows the numbers of TZPs in control and CVW-treated COCs at 0, 22, 42 h during IVM. All middle lines in box plot indicate the median, the edges indicate the 25th/75th percentiles and the whiskers indicate the 10th/90th percentiles. n.s., not significant. ^∗∗^*P* < 0.01 (Student’s *t*-test). **(C)** Representative images of putative zygotes with different numbers of pronuclei (PN) (horizontal arrows) at 18 h after fertilization. Scale bar = 30 μm. **(D)** Percentage of putative zygotes with different numbers of PN in control and CVW-treated groups. Error bars represent the SD in five replicates. Different superscripts indicate *P* < 0.05 (Student’s *t*-test).

A previous study has found that the migration and exocytosis of CGs are achieved in pig IVM oocytes, in accordance with our results (Figures 3B,C), but an effective block to polyspermy is still not fully established after CG exocytosis (Wang et al., 1997). Another study has reported that the size of the perivitelline space is closely related to the incidence of polyspermy in mouse and rabbit oocytes (Ueno and Niimura, 2008; Yoshida and Niimura, 2011). Interestingly, compared to the oocytes matured *in vivo*, pig IVM oocytes have been proved to show a narrow perivitelline space and an incomplete TZP retraction on the zona pellucida (Yuan et al., 2017). Because TZPs are gap junctional communications between cumulus cells and oocytes (Yuan et al., 2017), we thus hypothesized that abnormal TZP retraction in IVM oocytes might leave the gaps for sperm penetration on the zona pellucida, thereby causing polyspermy in porcine fertilized oocytes. In addition, the CVW medium might overcome defective TZP retraction and polyspermy since more CVW-matured oocytes showed an expanded perivitelline space (Supplementary Figure S2D). To test this, we collected CVW-treated and control COCs at 0, 22, 42 h during IVM, and labeled the TZPs by phalloidin-FITC staining (Figure 4A). As previously described (Yuan et al., 2017), the number of TZPs in control COCs was gradually decreased. A similar downtrend was shown in CVW-treated COCs, and their TZPs (45.0 ± 18.5) were prominently disconnected compared to those in controls (56.0 ± 15.5) at the end of the maturation period (Figure 4B). To evaluate the relationship between TZP integrity and polyspermy, both groups were subjected to IVF, and their numbers of pronuclei were counted at 18 h after fertilization (Figures 4C,D). Here, we showed that the percentage of polyspermic zygotes was significantly decreased from 59.5% in the control group to 43.0% in the experimental group, indicating that CVW-matured oocytes contained a more intact and functional zona pellucida with reduced TZPs to prevent polyspermy.

### Supplementation of CVW in IVM Medium Contributes to Cumulus Expansion

**FIGURE 5:**
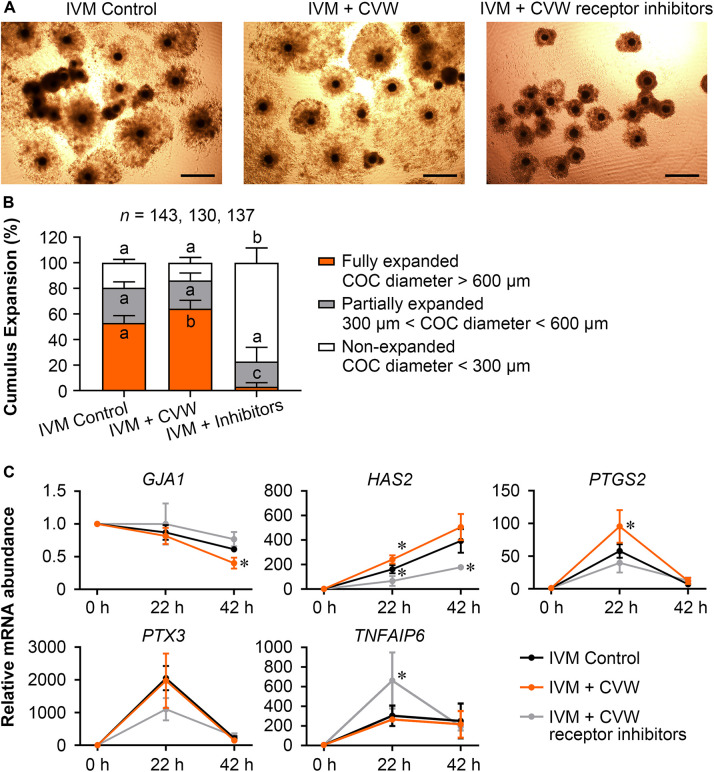
Effects of CVW and their receptor inhibitors on cumulus expansion. **(A)** Representative images of cumulus expansion in control, CVW-treated, and inhibitor-treated groups after IVM. Scale bar = 300 μm. **(B)** Percentage of COCs with different diameters in control, CVW-treated, and inhibitor-treated groups. Error bars represent the SD in five replicates. Different superscripts indicate *P* < 0.05 (one-way ANOVA with Duncan’s test). **(C)** Relative expression levels of cumulus expansion-related genes in control, CVW-treated, and inhibitor-treated groups at 0, 22, 42 h during IVM. The results from 0 h samples are set as 1, and are normalized to the internal control gene *GAPDH*. Error bars represent the SD in three replicates. ^∗^*P* < 0.05 (Student’s *t*-test).

Because TZP retraction could be regarded as a result of cumulus expansion (Suzuki et al., 2000), we next evaluated the expansion features of COCs treated with CVW and receptor inhibitors, including COC diameter and the expression of expansion-related genes (*GJA1*, *HAS2*, *PTGS2*, *PTX3*, and *TNFAIP6*) in cumulus cells (Kim et al., 2017; Zhang et al., 2018). As shown in Figures 5A,B, the majority (77.1%) of inhibitor-treated COCs displayed cumulus expansion failure (diameter < 300 μm) at 42 h of maturation. In contrast, the percentages of non-expanded COCs were 19.5 and 13.8% in control and CVW-treated groups, respectively. Importantly, there were more fully expanded COCs (64.2%, diameter > 600 μm) in IVM medium supplemented with CVW, compared to the controls (53.0%). To dissect the different cumulus expansion patterns in each group, we harvested cumulus cells at 0, 22, 42 h during IVM and investigated the abundance of expansion-related transcripts by qPCR. Downregulation of *GJA1*, which encodes a member of the connexin family, is consistent with the reduced communications between oocytes and cumulus cells (Kim et al., 2018). At 42 h, the lower abundance of *GJA1* mRNA represented a more complete TZP retraction in CVW-treated COCs compared to the controls (Figure 5C). Cumulus expansion also relies on the expression of *HAS2*, *PTGS2*, *PTX3*, and *TNFAIP6*, which are responsible for extracellular matrix establishment and organization during IVM (Park et al., 2004; Salustri et al., 2004). Here, compared to the control group, prominent upregulations of *HAS2* and *PTGS2* at 22 h might associate with more fully expanded COCs in the CVW-treated group (Figure 5C). Conversely, inhibitor treatment resulted in an unexpanded cumulus with aberrant expression of *HAS2* at 22 and 42 h, and *TNFAIP6* at 22 h of maturation (Figure 5C). The improved cumulus expansion in CVW-treated COCs suggests that CVW combination can promote oocyte maturation through cumulus cells as well. Meanwhile, the expansion failure in inhibitor-treated COCs also suggests a critical role of CVW receptor-mediated signals in this expansion process.

### Supplementation of CVW in IVM Medium Improves Oocyte Developmental Competence

**FIGURE 6:**
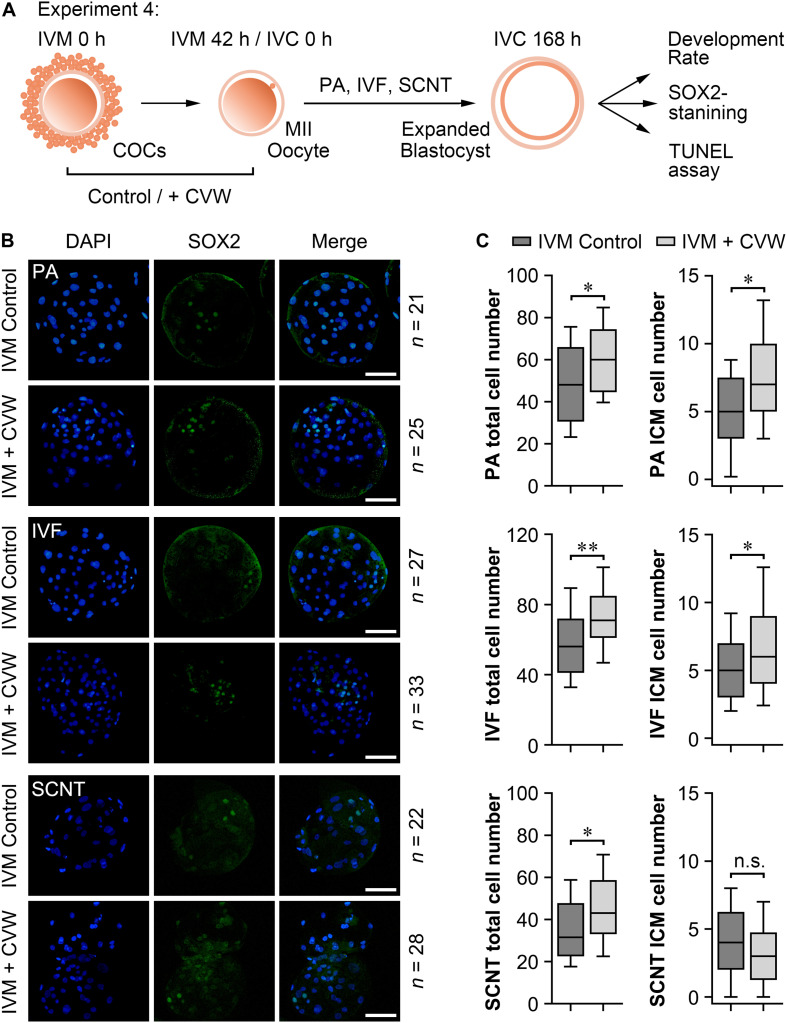
Cell numbers in blastocysts derived from oocytes matured with or without CVW supplementation. **(A)** Experimental design to determine the development rate and blastocyst quality. **(B)** Immunostaining of SOX2 (green) and DAPI (blue) in PA, IVF and SCNT blastocysts derived from control and CVW-treated oocytes. Scale bar = 100 μm. **(C)** Box plots show the total cell numbers and inner cell mass (ICM) cell numbers of PA, IVF and SCNT blastocysts derived from control and CVW-treated oocytes. All middle lines in box plots indicate the median, the edges indicate the 25th/75th percentiles and the whiskers indicate the 10th/90th percentiles. n.s, not significant. ^∗^*P* < 0.05, ^∗∗^*P* < 0.01 (Student’s *t*-test).

Subsequently, we examined the differences in developmental competence between oocytes matured with or without CVW supplementation. By performing PA, IVF, and SCNT procedures, the percentages of blastocyst development were counted after culture in unmodified PZM3 medium for 7 days (168 h) (Experiment 4, Figure 6A). Remarkably, in comparison with the control group (PA, 36.0%; IVF, 25.7%; SCNT, 18.4%), the efficiency of blastocyst formation showed a ∼10% elevation by using CVW-matured oocytes for PA (48.4%), IVF (34.9%), and SCNT (26.2%) (Table 1). Given that the total cell number, ICM cell number and apoptotic cell number are conventional criteria for the assessment of blastocyst quality (Liu et al., 2018b), we next measured these three criteria through SOX2 immunostaining (Bou et al., 2017) and TUNEL assay (Liu et al., 2018b) (Experiment 4, Figure 6A). Here, all the experimental groups (PA, 60.7 ± 16.3; IVF, 72.3 ± 19.3; SCNT, 45.9 ± 19.0) showed increased blastocyst cell numbers when compared to the control groups (PA, 49.1 ± 19.9; IVF, 58.0 ± 20.0; SCNT, 35.5 ± 16.5) (Figures 6B,C). Meanwhile, the ICM cell numbers were only elevated in PA (CVW, 7.5 ± 3.7; Control, 5.1 ± 2.9) and IVF (CVW, 6.9 ± 3.9; Control, 5.1 ± 2.8) blastocysts, but not in SCNT (CVW, 3.3 ± 2.7; Control, 4.0 ± 2.7) (Figures 6B,C). Furthermore, the use of CVW medium for maturation did not lead to the reduction of apoptotic cells in each group (PA, 3.2 ± 1.9; IVF, 3.2 ± 2.1; SCNT, 4.1 ± 2.2) when compared to the control groups (PA, 3.9 ± 2.0; IVF, 2.7 ± 2.0; SCNT, 4.6 ± 2.5) (Supplementary Figures S3A,B). Nevertheless, the apoptotic rates, calculated as the ratio of apoptotic cell numbers to total cell numbers, were significantly decreased in PA (CVW, 5.8 ± 3.2%; Control, 9.4 ± 5.8%) and SCNT (CVW, 10.5 ± 6.0%; Control, 13.8 ± 6.4%) blastocysts, but not in IVF (CVW, 4.8 ± 3.6%; Control, 5.0 ± 2.9%) (Supplementary Figure S3B). Overall, these results imply that CVW-matured oocytes hold higher potential to develop to the blastocyst stage, and the embryo quality is also partially improved.

**TABLE 1:** Effects of combined CVW supplementation on porcine embryonic development *in vitro*.

Group	IVM medium supplementation*	Number of oocytes	Cleaved per oocyte (%)^‡^	Blastocyst per oocyte (%)^‡^
PA	CVW (–)	265	82.1 ± 4.3	36.0 ± 4.2^a^
	CVW (+)	277	86.1 ± 3.5	48.4 ± 5.0^b^
IVF	CVW (–)	292	60.8 ± 4.2	25.7 ± 3.8^a^
	CVW (+)	306	63.7 ± 2.1	34.9 ± 2.2^b^
SCNT	CVW (–)	254	57.0 ± 3.3	18.4 ± 4.8^a^
	CVW (+)	287	53.9 ± 2.2	26.2 ± 3.7^b^

*CVW (–), IVM medium without CVW supplementation; CVW (+), IVM medium with CVW supplementation. ^‡^Data are shown as the mean percentage ± SD in six replicates. ^*a,b*^Values with different superscripts differ significantly within the same column from each other (*P* < 0.05, Student’s *t*-test).

### Supplementation of CVW Modulates MAPK and Canonical WNT Signaling Pathways During IVM

**FIGURE 7:**
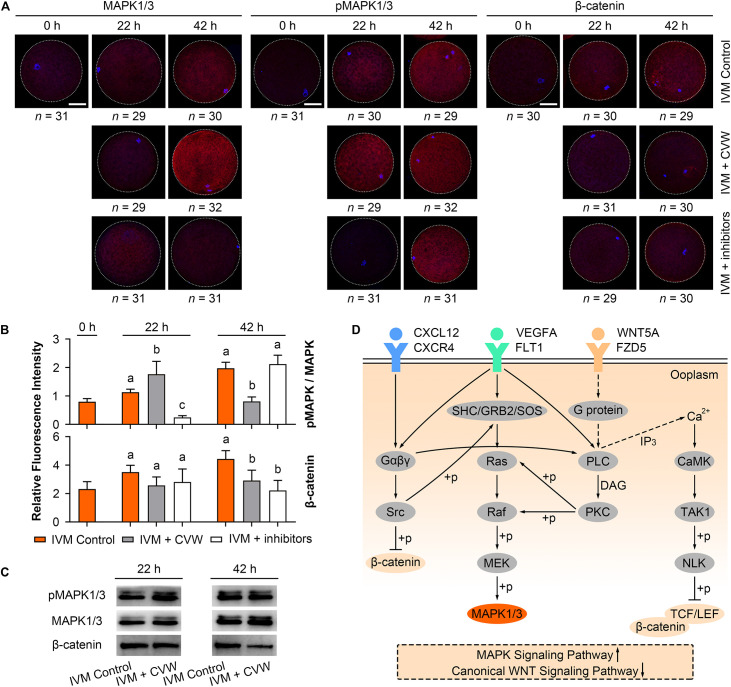
Effects of CVW and their receptor inhibitors on MAPK and canonical WNT pathway during IVM. **(A)** Immunostaining of MAPK1/3, phosphorylated MAPK1/3 (pMAPK1/3), and β-catenin (red) and DAPI (blue) in control, CVW-treated, and inhibitor-treated oocytes at 0, 22, 42 h during IVM. Scale bar = 30 μm. **(B)** Bar plots show the fluorescence intensities of pMAPK/MAPK and β-catenin in control, CVW-treated, and inhibitor-treated oocytes at 0, 22, 42 h during IVM. Error bars represent the SD in three replicates. Different superscripts indicate *P* < 0.05 (one-way ANOVA with Duncan’s test). **(C)** Representative images of immunoblots showing the signal intensities of MAPK, pMAPK and β-catenin in control and CVW-treated oocytes at 22 and 42 h during IVM. **(D)** Schematic representation of the proposed mechanism illustrating the interactions between CVW and their receptors on the oolemma, as well as the downstream signaling pathways regulated by CVW. Signal transduction pathways are summarized from the KEGG database (https://www.kegg.jp/). Dashed arrows stand for putative interactions. Oocyte maturation improvement by CVW is achieved through activating the MAPK pathway, as well as inhibiting the canonical WNT pathway during IVM.

During IVM, elevated MAPK activity has been proved to be necessary for oocyte meiotic maturation (Yan et al., 2012; Mo et al., 2014; Lopez-Cardona et al., 2017), while canonical WNT signals are stronger in oocytes matured *in vitro* compared to *in vivo* (Spate et al., 2014). To check if CVW and their receptor inhibitors would affect the MAPK and canonical WNT signaling pathways, we initially performed immunofluorescence assays and measured the fluorescence intensity of each pathway in oocytes. As shown in Figures 7A,B, in the control oocytes, the ratio of phosphorylated MAPK1/3 (pMAPK1/3) to total MAPK1/3 was enhanced during IVM. By contrast, relative pMAPK1/3 signals were apparently stronger in CVW-treated oocytes at 22 h of maturation, and declined to a lower level compared to the controls at 42 h (Figures 7A,B). Meanwhile, in the presence of CVW receptor inhibitors, MAPK1/3 phosphorylation was blocked at 22 h, and then elevated at a comparable level to the controls at 42 h (Figures 7A,B). As for canonical WNT pathways, a gradually increasing level of β-catenin was determined in the control group during the whole IVM period (Figures 7A,B). Conversely, β-catenin abundance exhibited a slight upregulation when oocytes were exposed to CVW cytokines, which was prominently lower compared to those in controls at 42 h (Figures 7A,B). For some unknown reason, when compared to CVW-treated oocytes, inhibition of CVW receptor functions remained at a similarly low catenin expression at 22 h, and this expression was moderately decreased at 42 h (Figures 7A,B). Next, the immunostaining results were further confirmed via immunoblotting, as the pMAPK1/3 protein in CVW-matured oocytes was more abundant than in the control group at 22 h, while the expression of β-catenin was obviously reduced in CVW-matured oocytes at 42 h (Figure 7C and Supplementary Figure S4). These results indicate that CVW combination can provide much improved oocyte competence through activating the MAPK pathway in advance, as well as inhibiting the canonical WNT pathway at the end of the IVM period. A possible synergistic modulation of CVW-mediated signaling pathways is shown in Figure 7D.

## Discussion

Establishment and optimization of the oocyte IVM system is a critical step for assisted reproductive technologies. To our knowledge, the effect of WNT5A on oocyte maturation has never been reported, while the elevating effect of VEGFA has been determined in porcine (Kere et al., 2014), bovine (Luo et al., 2002), and ovine (Yan et al., 2012) oocytes. A previous study has demonstrated that the maturation efficiency is only increased in porcine denuded oocytes at 5 ng/mL of VEGFA, but not in COCs (Kere et al., 2014). However, in our TCM-199-based IVM medium, we could observe an obvious improvement when oocytes were cultured with intact cumulus cells at 10 and 25 ng/mL of VEGFA (Figures 2A,B), and both concentrations were not measured in previous study (Kere et al., 2014). Recently, a novel role of CXCL12 in regulating meiotic maturation was uncovered in ovine oocytes, and this was achieved via cumulus cells even though CXCR4 was expressed in oocytes throughout the whole follicle growth stage (Zhang et al., 2018). As for the pig, our results revealed that 10 ng/mL CXCL12 could promote denuded oocytes to extrude the PB1 (Figures 2A,B), suggesting that a direct CXCL12-CXCR4 interaction occurred during porcine IVM. In fact, all of these CVW factors have been detected in human and bovine granulosa cells (Skinner et al., 2008; Nishigaki et al., 2011; Sanchez et al., 2014; Abedini et al., 2015). The concentrations of CXCL12 and VEGFA in human follicular fluid are enriched with a positive correlation during follicle development (Nishigaki et al., 2011), while conditional Wnt5a-knockout mice exhibit follicular atresia and ovulation failure (Abedini et al., 2016). These findings indicate that our CVW supplementation may ultimately improve IVM outcome in humans and other species.

In this paper, the CVW medium showed a ∼20% elevation in porcine oocyte maturation (Figure 2C), which is lower compared to the beneficial effects of the FLI medium (∼35%) (Yuan et al., 2017). One explanation is that the components of these two culture systems are totally different. The FLI medium is a chemically defined medium, while our system is supplemented with 10% follicular fluid, which contains oocyte maturation inhibitors, such as hypoxanthine (Downs et al., 1985). It should be noted that follicular fluid is indispensable in the CVW medium, since it would cause unexpanded COCs and maturation block when cultured without this fluid (data not shown). Intriguingly, when COCs were cultured in our medium with FLI addition (FGF2, 40 ng/mL; LIF, 20 ng/mL; IGF1, 20 ng/mL) (Yuan et al., 2017), no maturation improvement (62.2%) was observed compared to the controls (58.6%) (Supplementary Figure S2E). It suggests that at least CVW combination is applicable in the current IVM medium that we used. Nevertheless, future studies should confirm the effects of CVW on oocyte maturation by using a chemically defined medium. Another difference between FLI and CVW media is the approach to achieving maturation. FLI combination provides improved oocyte competence mainly through an indirect way, such as promoting cumulus expansion and TZP retraction (Yuan et al., 2017). By contrast, CVW combination not only promotes cumulus expansion (Figure 5) and TZP retraction (Figures 4A,B) in COCs, but also improves denuded oocyte maturation (Figure 2D), indicating that a direct effect occurred on the oocyte itself. Moreover, by using the FLI medium, it has revealed the relationship between the size of perivitelline space and TZP integrity (Yuan et al., 2017). Here, we further associated both of them with polyspermic penetration in CVW-matured oocytes (Figures 4C,D). The results could be explained in that cumulus and perivitelline space expansions are necessary for severing the connections between oocytes and cumulus cells, thus closing the channels for sperm penetration on the zona pellucida, and reducing the incidence of polyspermy after IVF.

Inhibitor utilization also provides evidence that CXCL12-CXCR4, VEGFA-FLT1, and WNT5A-FZD5 interactions are critical for porcine oocyte maturation, as an apparent meiotic impairment could be seen when these interactions were inhibited individually (Supplementary Figure S2B) or in combination (Figure 2D). Notably, in the FLI medium, individual inhibition of FGF2- and LIF-mediated signals exhibited no influence on nuclear maturation when compared to the controls; only IGF1 receptor inhibitor could significantly reduce the maturation rate (Yuan et al., 2017). The expression level of each corresponding receptor might cause this difference, as the mRNA abundances of CVW and IGF1 receptor genes were relatively higher compared to FGF2 and LIF receptors in porcine oocytes (Supplementary Figure S5 and Supplementary Table S2). Additionally, CVW receptor inhibition was detrimental to PA blastocyst formation (Supplementary Figure S2B), suggesting that the signaling pathways activated by CVW receptors are critical for porcine preimplantation development as well. Combined with the highly expressed CVW factors in reproductive tissues (Figure 1A), these results encourage us to modify the IVC medium for pig embryos through CVW supplementation. However, we could only find an obvious elevation of the porcine PA blastocyst rate when PZM3 medium was supplemented with VEGFA, in accordance with previous studies (Biswas et al., 2011; Kere et al., 2014), but not in CXCL12- and WNT5A-treated groups (Supplementary Figure S2A). We noticed that the application time might affect blastocyst production *in vitro*, since the effect of VEGFA on SCNT embryos was prominently different when it was added during the early stages (days 1–3) or the late stages (days 4–7) (Biswas et al., 2011). Therefore, the precise and synergistic effects of CVW factors in PZM3 medium will require further investigation.

Finally, the IVM outcome of CVW supplementation can be elucidated by cell signaling changes. It is well known that MAPK activation can promote meiotic resumption, germinal vesicle breakdown and spindle microtubule organization (Yu et al., 2007; Schindler, 2011). A steady rise of MAPK phosphorylation has been revealed in mouse (Lopez-Cardona et al., 2017), bovine (Mo et al., 2014) and ovine (Yan et al., 2012) oocytes during IVM. Similarly, porcine oocytes exhibited an elevating ratio of pMAPK to MAPK in control maturation medium, but this ratio was highly increased at 22 h and then decreased in the CVW medium (Figures 7A–C and Supplementary Figure S4). Given that the reduction of MAPK activity is nesserary for MII stage exit and oocyte activation (Moos et al., 1996; Phillips et al., 2002), the dephosphorylation process at 42 h might imply that CVW-matured oocytes have already prepared for subsequent preimplantation development. Indeed, *in vitro* production of PA, IVF and SCNT blastocysts became more efficient by using CVW-matured oocytes (Table 1). Furthermore, we determined a decreased abundance of β-catenin in CVW-matured oocytes at the end of the IVM period (Figures 7A–C and Supplementary Figure S4), suggesting that the improved oocyte competence may be related to canonical WNT pathway inhibition as well. In fact, overrepresented WNT signals have been examined in porcine IVM oocytes (Spate et al., 2014), while addition of Dickkopf-related protein 1 (DKK1) (Spate et al., 2014) or compound FH535 (Shi et al., 2018) could weaken these signals to promote nuclear maturation. Here, we speculate that CVW may stimulate calcium signaling to promote the degradation of β-catenin coactivators, T cell factor (TCF) and lymphoid enhancer binding factor (LEF), or directly result in phosphorylation and lysis of β-catenin by non-receptor tyrosine kinase SRC and proteasomes, respectively (Figure 7D). A detailed cell signaling network remains to be explored since CVW may also activate other pathways during IVM, such as PI3K-Akt pathway, Jak-STAT pathway, and p38 MAPK pathway.

## Conclusion

In conclusion, we provided a new combination of three maternal cytokines, CXCL12, VEGFA, and WNT5A, for improving porcine oocyte maturation and its subsequent embryonic development. On the one hand, CVW supplementation could directly bind to their receptors on the oolemma, modulate the downstream MAPK and WNT signals in the ooplasm, thus endowing the oocyte with a better developmental competence to reach the blastocyst stage. On the other hand, CVW factors displayed beneficial effects on cumulus expansion, thus allowing TZPs to retract more completely from the zona pellucida, which contributes to preventing polyspermic penetration in fertilized oocytes. Subsequent studies are required to determine the effects of CVW supplementation on piglet production through IVF and SCNT embryo transfer. These observations not only advance the understanding of the mammalian oocyte maturation process, but also provide a more precise approach to recapitulating the maternal environment in our IVM culture system, thereby improving IVM outcome for assisted reproduction.

## Data Availability Statement

Publicly available datasets were analyzed in this study. This data can be found here: Gene Expression Omnibus database (https://www.ncbi.nlm.nih.gov/geo/) under accession numbers GSE139512 and GSE108570.

## Ethics Statement

The animal study was reviewed and approved by Animal Care and Use Committee of Huazhong Agriculture University, Wuhan, China.

## Author Contributions

XL and Y-LM conceived and designed the research. YH, ZKL, GB, ZTL, and XH collected the samples and performed the experiments. XL, JZ, and HZ analyzed the RNA-seq and experimental data. XL and YH wrote the original manuscript. XZ and Y-LM revised the manuscript. All authors read and approved the final version of the manuscript.

## Conflict of Interest

The authors declare that the research was conducted in the absence of any commercial or financial relationships that could be construed as a potential conflict of interest.

## Footnotes

1 https://www.ncbi.nlm.nih.gov/geo/

2 https://www.kegg.jp/

3 https://meshb.nlm.nih.gov

## References

[B1] Abedini A. Zamberlam G. Boerboom D. Price C. A. (2015). Non-canonical WNT5A is a potential regulator of granulosa cell function in cattle. *Mol. Cell Endocrinol.* 403 39–45. 10.1016/j.mce.2015.01.017 25600632

[B2] Abedini A. Zamberlam G. Lapointe E. Tourigny C. Boyer A. Paquet M. (2016). WNT5a is required for normal ovarian follicle development and antagonizes gonadotropin responsiveness in granulosa cells by suppressing canonical WNT signaling. *FASEB J.* 30 1534–1547. 10.1096/fj.15-280313 26667040 PMC4799500

[B3] An L. Liu J. Du Y. Liu Z. Zhang F. Liu Y. (2018). Synergistic effect of cysteamine, leukemia inhibitory factor, and Y27632 on goat oocyte maturation and embryo development in vitro. *Theriogenology* 108 56–62. 10.1016/j.theriogenology.2017.11.028 29197293

[B4] Biswas D. Jung E. M. Jeung E. B. Hyun S. H. (2011). Effects of vascular endothelial growth factor on porcine preimplantation embryos produced by in vitro fertilization and somatic cell nuclear transfer. *Theriogenology* 75 256–267. 10.1016/j.theriogenology.2010.08.012 20961610

[B5] Bou G. Liu S. Sun M. Zhu J. Xue B. Guo J. (2017). CDX2 is essential for cell proliferation and polarity in porcine blastocysts. *Development* 144 1296–1306. 10.1242/dev.141085 28219949

[B6] Dang-Nguyen T. Q. Haraguchi S. Kikuchi K. Somfai T. Bodo S. Nagai T. (2014). Leukemia inhibitory factor promotes porcine oocyte maturation and is accompanied by activation of signal transducer and activator of transcription 3. *Mol. Reprod. Dev.* 81 230–239. 10.1002/mrd.22289 24307388

[B7] Downs S. M. Coleman D. L. Ward-Bailey P. F. Eppig J. J. (1985). Hypoxanthine is the principal inhibitor of murine oocyte maturation in a low molecular weight fraction of porcine follicular fluid. *Proc. Natl. Acad. Sci. U.S.A.* 82 454–458. 10.1073/pnas.82.2.454 2982158 PMC397057

[B8] Jenei V. Sherwood V. Howlin J. Linnskog R. Safholm A. Axelsson L. (2009). A t-butyloxycarbonyl-modified Wnt5a-derived hexapeptide functions as a potent antagonist of Wnt5a-dependent melanoma cell invasion. *Proc. Natl. Acad. Sci. U.S.A.* 106 19473–19478. 10.1073/pnas.0909409106 19901340 PMC2780806

[B9] Kere M. Siriboon C. Liao J. W. Lo N. W. Chiang H. I. Fan Y. K. (2014). Vascular endothelial growth factor A improves quality of matured porcine oocytes and developing parthenotes. *Domest. Anim. Endocrinol.* 49 60–69. 10.1016/j.domaniend.2014.06.002 25061966

[B10] Kiapekou E. Loutradis D. Drakakis P. Zapanti E. Mastorakos G. Antsaklis A. (2005). Effects of GH and IGF-I on the in vitro maturation of mouse oocytes. *Hormones* 4 155–160. 10.14310/horm.2002.11153 16613825

[B11] Kim D. H. Lee H. R. Kim M. G. Lee J. S. Jin S. J. Lee H. T. (2017). The effect of poly(ADP-ribosyl)ation inhibition on the porcine cumulus-oocyte complex during in vitro maturation. *Biochem. Biophys. Res. Commun.* 483 752–758. 10.1016/j.bbrc.2016.12.070 27965086

[B12] Kim J. M. Park J. E. Yoo I. Han J. Kim N. Lim W. J. (2018). Integrated transcriptomes throughout swine oestrous cycle reveal dynamic changes in reproductive tissues interacting networks. *Sci. Rep.* 8:5436. 10.1038/s41598-018-23655-1 29615657 PMC5882957

[B13] Kong Q. Xie B. Li J. Huan Y. Huang T. Wei R. (2014). Identification and characterization of an oocyte factor required for porcine nuclear reprogramming. *J. Biol. Chem.* 289 6960–6968. 10.1074/jbc.M113.543793 24474691 PMC3945357

[B14] Kong Q. Yang X. Zhang H. Liu S. Zhao J. Zhang J. (2020). Lineage specification and pluripotency revealed by transcriptome analysis from oocyte to blastocyst in pig. *FASEB J.* 34 691–705. 10.1096/fj.201901818RR 31914626

[B15] Lai L. Prather R. S. (2003). Production of cloned pigs by using somatic cells as donors. *Cloning Stem Cells* 5 233–241. 10.1089/153623003772032754 14733743

[B16] Liu X. Nie Z. W. Gao Y. Y. Chen L. Yin S. Y. Zhang X. (2018a). Sodium fluoride disturbs DNA methylation of NNAT and declines oocyte quality by impairing glucose transport in porcine oocytes. *Environ. Mol. Mutagen.* 59 223–233. 10.1002/em.22165 29285797

[B17] Liu X. Wang Y. Gao Y. Su J. Zhang J. Xing X. (2018b). H3K9 demethylase KDM4E is an epigenetic regulator for bovine embryonic development and a defective factor for nuclear reprogramming. *Development* 145:dev158261. 10.1242/dev.158261 29453221

[B18] Lonergan P. Carolan C. Van Langendonckt A. Donnay I. Khatir H. Mermillod P. (1996). Role of epidermal growth factor in bovine oocyte maturation and preimplantation embryo development in vitro. *Biol. Reprod.* 54 1420–1429. 10.1095/biolreprod54.6.1420 8724373

[B19] Lopez-Cardona A. P. Perez-Cerezales S. Fernandez-Gonzalez R. Laguna-Barraza R. Pericuesta E. Agirregoitia N. (2017). CB1 cannabinoid receptor drives oocyte maturation and embryo development via PI3K/Akt and MAPK pathways. *FASEB J.* 31 3372–3382. 10.1096/fj.201601382RR 28428264

[B20] Luo H. Kimura K. Aoki M. Hirako M. (2002). Effect of vascular endothelial growth factor on maturation, fertilization and developmental competence of bovine oocytes. *J. Vet. Med. Sci.* 64 803–806. 10.1292/jvms.64.803 12399605

[B21] Mo X. Wu G. Yuan D. Jia B. Liu C. Zhu S. (2014). Leukemia inhibitory factor enhances bovine oocyte maturation and early embryo development. *Mol. Reprod. Dev.* 81 608–618. 10.1002/mrd.22327 24687528

[B22] Moos J. Kopf G. S. Schultz R. M. (1996). Cycloheximide-induced activation of mouse eggs: effects on cdc2/cyclin B and MAP kinase activities. *J. Cell Sci.* 109 739–748. 8718665 10.1242/jcs.109.4.739

[B23] Nie Z. W. Chen L. Jin Q. S. Gao Y. Y. Wang T. Zhang X. (2017). Function and regulation mechanism of Chk1 during meiotic maturation in porcine oocytes. *Cell Cycle* 16 2220–2229. 10.1080/15384101.2017.1373221 28933982 PMC5736331

[B24] Nishigaki A. Okada H. Okamoto R. Sugiyama S. Miyazaki K. Yasuda K. (2011). Concentrations of stromal cell-derived factor-1 and vascular endothelial growth factor in relation to the diameter of human follicles. *Fertil. Steril.* 95 742–746. 10.1016/j.fertnstert.2010.10.028 21071025

[B25] Niu D. Wei H. J. Lin L. George H. Wang T. Lee I. H. (2017). Inactivation of porcine endogenous retrovirus in pigs using CRISPR-Cas9. *Science* 357 1303–1307. 10.1126/science.aan4187 28798043 PMC5813284

[B26] Oberlender G. Murgas L. D. Zangeronimo M. G. da Silva A. C. Menezes Tde A. Pontelo T. P. (2013). Role of insulin-like growth factor-I and follicular fluid from ovarian follicles with different diameters on porcine oocyte maturation and fertilization in vitro. *Theriogenology* 80 319–327. 10.1016/j.theriogenology.2013.04.018 23683690

[B27] Park J. Y. Su Y. Q. Ariga M. Law E. Jin S. L. Conti M. (2004). EGF-like growth factors as mediators of LH action in the ovulatory follicle. *Science* 303 682–684. 10.1126/science.1092463 14726596

[B28] Pereira G. R. Lorenzo P. L. Carneiro G. F. Ball B. A. Goncalves P. B. Pegoraro L. M. (2012). The effect of growth hormone (GH) and insulin-like growth factor-I (IGF-I) on in vitro maturation of equine oocytes. *Zygote* 20 353–360. 10.1017/S0967199411000335 21794202

[B29] Phillips K. P. Petrunewich M. A. Collins J. L. Booth R. A. Liu X. J. Baltz J. M. (2002). Inhibition of MEK or cdc2 kinase parthenogenetically activates mouse eggs and yields the same phenotypes as Mos(-/-) parthenogenotes. *Dev. Biol.* 247 210–223. 10.1006/dbio.2002.0680 12074563

[B30] Prochazka R. Srsen V. Nagyova E. Miyano T. Flechon J. E. (2000). Developmental regulation of effect of epidermal growth factor on porcine oocyte-cumulus cell complexes: nuclear maturation, expansion, and F-actin remodeling. *Mol. Reprod. Dev.* 56 63–73. 10.1002/(sici)1098-2795(200005)56:1<63::aid-mrd8>3.0.co;2-d 10737968

[B31] Romar R. Funahashi H. Coy P. (2016). In vitro fertilization in pigs: new molecules and protocols to consider in the forthcoming years. *Theriogenology* 85 125–134. 10.1016/j.theriogenology.2015.07.017 26271164

[B32] Ruan D. Peng J. Wang X. Ouyang Z. Zou Q. Yang Y. (2018). XIST derepression in active X chromosome hinders pig somatic cell nuclear transfer. *Stem Cell Rep.* 10 494–508. 10.1016/j.stemcr.2017.12.015 29337117 PMC5830944

[B33] Salustri A. Garlanda C. Hirsch E. De Acetis M. Maccagno A. Bottazzi B. (2004). PTX3 plays a key role in the organization of the cumulus oophorus extracellular matrix and in in vivo fertilization. *Development* 131 1577–1586. 10.1242/dev.01056 14998931

[B34] Sanchez A. M. Vigano P. Quattrone F. Pagliardini L. Papaleo E. Candiani M. (2014). The WNT/beta-catenin signaling pathway and expression of survival promoting genes in luteinized granulosa cells: endometriosis as a paradigm for a dysregulated apoptosis pathway. *Fertil. Steril.* 101 1688–1696. 10.1016/j.fertnstert.2014.02.040 24661731

[B35] Schindler K. (2011). Protein kinases and protein phosphatases that regulate meiotic maturation in mouse oocytes. *Results Probl. Cell Differ.* 53 309–341. 10.1007/978-3-642-19065-0_14 21630151

[B36] Shi M. Cheng J. He Y. Jiang Z. Bodinga B. M. Liu B. (2018). Effect of FH535 on in vitro maturation of porcine oocytes by inhibiting WNT signaling pathway. *Anim. Sci. J.* 89 631–639. 10.1111/asj.12982 29284185

[B37] Skinner M. K. Schmidt M. Savenkova M. I. Sadler-Riggleman I. Nilsson E. E. (2008). Regulation of granulosa and theca cell transcriptomes during ovarian antral follicle development. *Mol. Reprod. Dev.* 75 1457–1472. 10.1002/mrd.20883 18288646 PMC5749411

[B38] Spate L. D. Brown A. N. Redel B. K. Whitworth K. M. Murphy C. N. Prather R. S. (2014). Dickkopf-related protein 1 inhibits the WNT signaling pathway and improves pig oocyte maturation. *PLoS One* 9:e95114. 10.1371/journal.pone.0095114 24739947 PMC3989281

[B39] Suzuki H. Jeong B. S. Yang X. (2000). Dynamic changes of cumulus-oocyte cell communication during in vitro maturation of porcine oocytes. *Biol. Reprod.* 63 723–729. 10.1095/biolreprod63.3.723 10952913

[B40] Ueno S. Niimura S. (2008). Size of perivitelline space and incidence of polyspermy in mouse oocytes matured in vivo and in vitro. *J. Mammal. Ova. Res.* 25 44–49. 10.1274/jmor.25.44

[B41] Valleh M. V. Zandi N. K. Rasmussen M. A. Hyttel P. (2017). Optimal doses of EGF and GDNF act as biological response modifiers to improve porcine oocyte maturation and quality. *Zygote* 25 423–433. 10.1017/S0967199417000181 28693648

[B42] Wang T. Gao Y. Y. Chen L. Nie Z. W. Cheng W. Liu X. (2017). Melatonin prevents postovulatory oocyte aging and promotes subsequent embryonic development in the pig. *Aging* 9 1552–1564. 10.18632/aging.101252 28657543 PMC5509455

[B43] Wang W. H. Sun Q. Y. Hosoe M. Shioya Y. Day B. N. (1997). Quantified analysis of cortical granule distribution and exocytosis of porcine oocytes during meiotic maturation and activation. *Biol. Reprod.* 56 1376–1382. 10.1095/biolreprod56.6.1376 9166688

[B44] Yan L. Luo H. Gao X. Liu K. Zhang Y. (2012). Vascular endothelial growth factor-induced expression of its receptors and activation of the MAPK signaling pathway during ovine oocyte maturation in vitro. *Theriogenology* 78 1350–1360. 10.1016/j.theriogenology.2012.06.001 22898011

[B45] Yan S. Tu Z. Liu Z. Fan N. Yang H. Yang S. (2018). A huntingtin knockin pig model recapitulates features of selective neurodegeneration in Huntington’s Disease. *Cell* 173 989.e13–1002.e13. 10.1016/j.cell.2018.03.005 29606351 PMC5935586

[B46] Yoshida N. Niimura S. (2011). Size of the perivitelline space and incidence of polyspermy in rabbit and hamster oocytes. *Reprod. Med. Biol.* 10 31–41. 10.1007/s12522-010-0067-0 29662352 PMC5892974

[B47] Yu L. Z. Xiong B. Gao W. X. Wang C. M. Zhong Z. S. Huo L. J. (2007). MEK1/2 regulates microtubule organization, spindle pole tethering and asymmetric division during mouse oocyte meiotic maturation. *Cell Cycle* 6 330–338. 10.4161/cc.6.3.3805 17297311

[B48] Yuan Y. Spate L. D. Redel B. K. Tian Y. Zhou J. Prather R. S. (2017). Quadrupling efficiency in production of genetically modified pigs through improved oocyte maturation. *Proc. Natl. Acad. Sci. U.S.A.* 114 E5796–E5804. 10.1073/pnas.1703998114 28673989 PMC5530680

[B49] Zhang R. N. Pang B. Xu S. R. Wan P. C. Guo S. C. Ji H. Z. (2018). The CXCL12-CXCR4 signaling promotes oocyte maturation by regulating cumulus expansion in sheep. *Theriogenology* 107 85–94. 10.1016/j.theriogenology.2017.10.039 29132039

[B50] Zheng Q. Lin J. Huang J. Zhang H. Zhang R. Zhang X. (2017). Reconstitution of UCP1 using CRISPR/Cas9 in the white adipose tissue of pigs decreases fat deposition and improves thermogenic capacity. *Proc. Natl. Acad. Sci. U.S.A.* 114 E9474–E9482. 10.1073/pnas.1707853114 29078316 PMC5692550

