# Supplementary material for: Maternal Cytokines CXCL12, VEGFA, and WNT5A Promote Porcine Oocyte Maturation via MAPK Activation and Canonical WNT Inhibition
Source: Front Cell Dev Biol. 2020 Jul 7;8:578. doi: 10.3389/fcell.2020.00578 (PMC7358312; doi:10.3389/fcell.2020.00578)
Supplement: Supplementary file 1 [file Data_Sheet_1.PDF]

Supplementary Material

1 Supplementary Tables

Table S1. Sequences of quantitative PCR primers.

Gene Name	Accession Number	Sense (5′ to 3′)	Antisense (5′ to 3′)
GJA1	NM_001244212	ACTGAGCCCCTCCAAAGACT	GCTCGGCACTGTAATTAGCC
HAS2	NM_214053	GAAGTCATGGGCAGGGACAATTC	TGGCAGGCCCTTTCTATGTTA
PTGS2	NM_214321	TCGACCAGAGCAGAGAGATGAGAT	ACCATAGAGCGCTTCTAACTCTGC
PTX3	NM_001244783	AGACTTTATGCCATGGTGCT	TGACAGTGAGCAATGAACAA
TNFAIP6	NM_001159607	TACGACAGTTACGACGACGT	CACGGAAGCATCACTTAGGA
GAPDH	NM_001206359	CAAGGGCATCCTGGGCTACAC	GGATCGAGTTGGGGCTGTGAC

Table S2. Expression levels of intercellular signaling ligands and receptors in pig oocytes, embryos and reproductive tissues.

See supplementary Excel.

2 Supplementary Figures

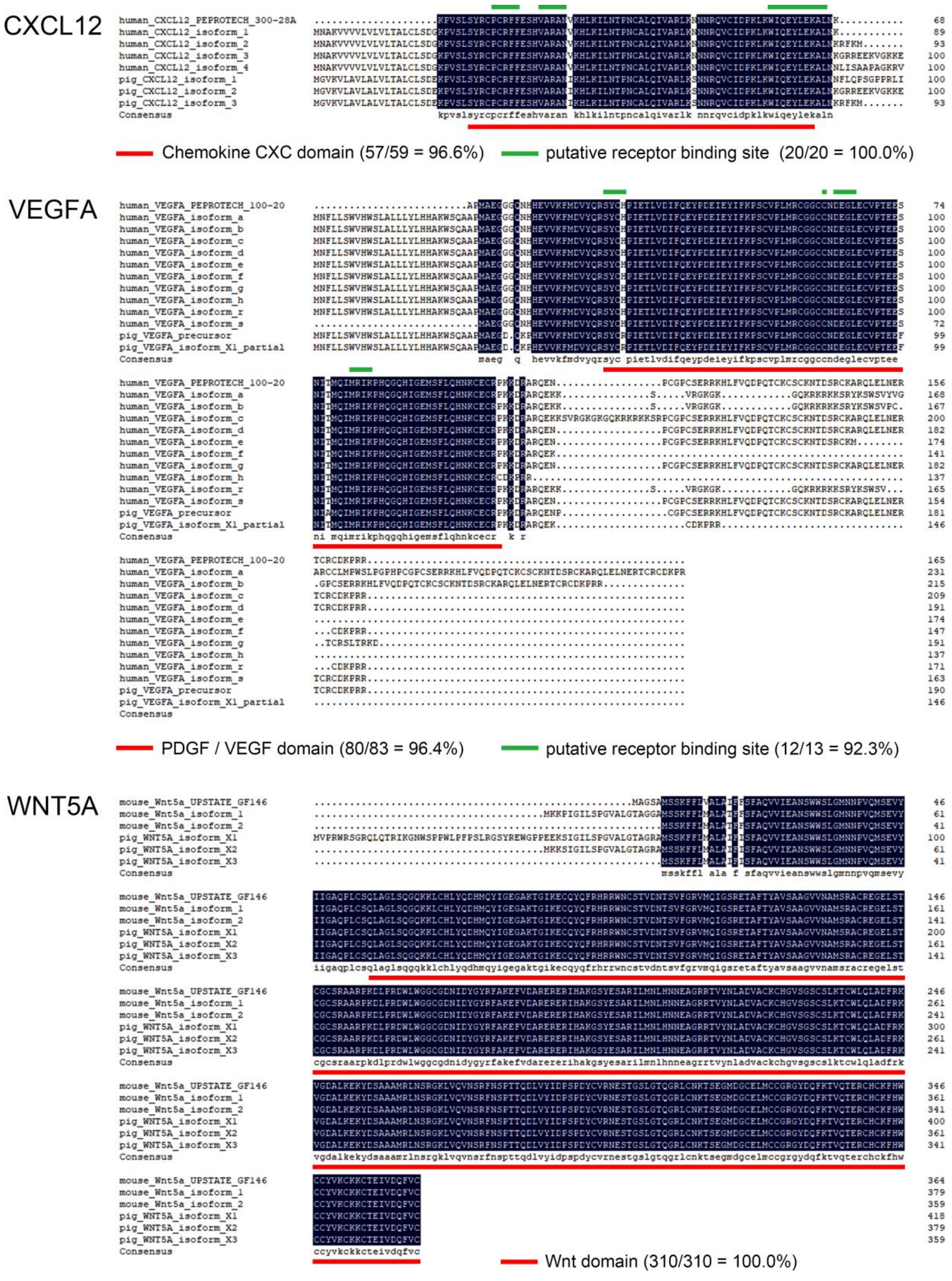

Supplementary Figure 1. Protein sequence alignment of CVW cytokines from porcine, mouse and human. 100% conserved amino acids are marked in dark blue. Functional protein domains and

putative receptor binding sites are summarized from the Pfam database (http://pfam.xfam.org/), and are marked in red (below) and green (above) bands, respectively. Sequence homologies are shown in parentheses.

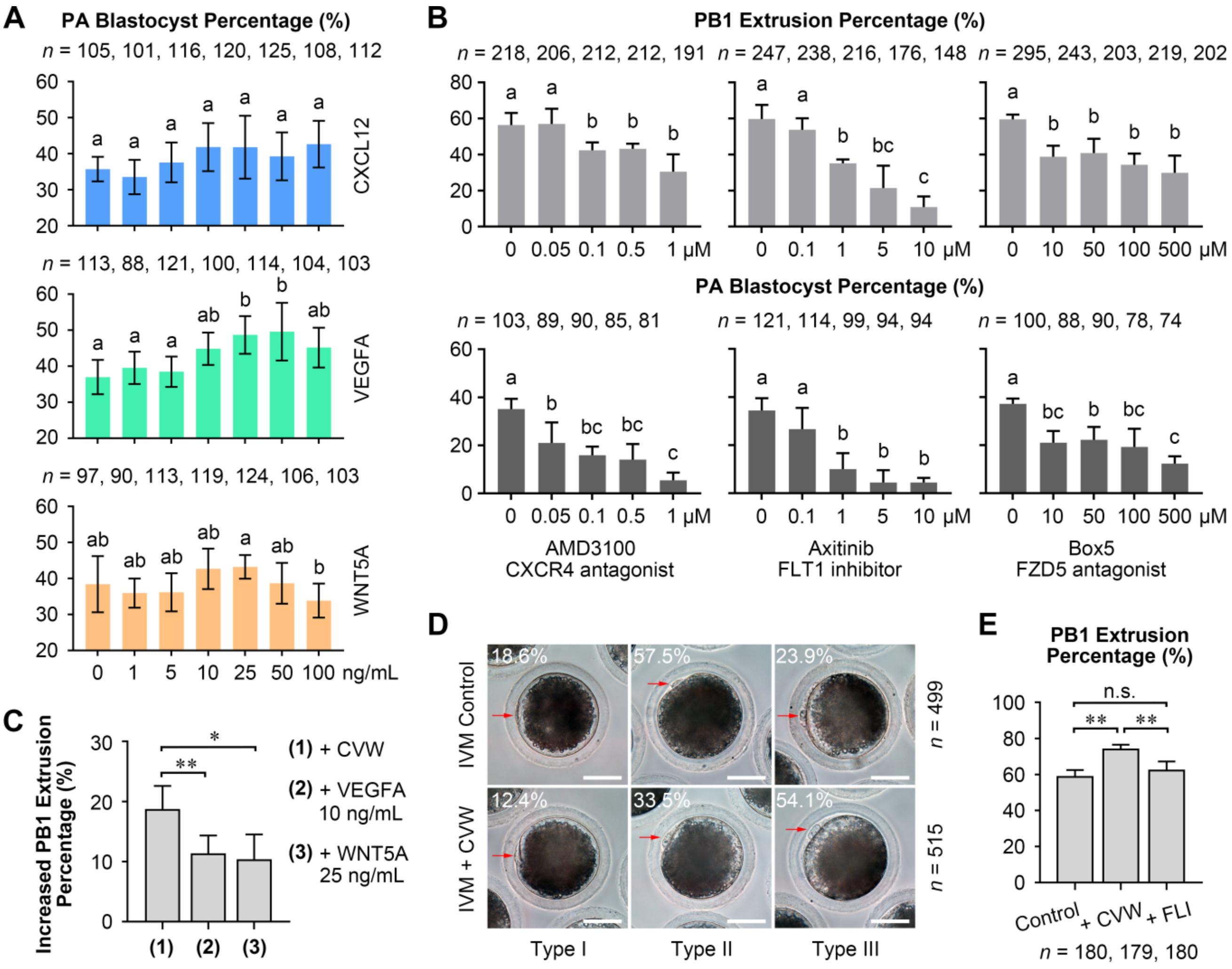

Supplementary Figure 2. Effects of CVW and their receptor inhibitors on porcine oocyte maturation and embryo development. **(A)** Individual effects of CVW on PA blastocyst formation when factors are added to IVC medium (PZM3). Error bars represent the SD in four replicates. Different superscripts indicate $P < 0.05$ (one-way ANOVA with Duncan's test). **(B)** Individual effects of CVW receptor inhibitors on oocyte PB1 extrusion (above) and PA blastocyst formation (below) when inhibitors are added to IVM and IVC media, respectively. Error bars represent the SD in three replicates. Different superscripts indicate $P < 0.05$ (one-way ANOVA with Duncan's test). **(C)** The increased percentage of PB1 extrusion via combined CVW treatment and individual treatment by 10 ng/mL VEGFA and 25 ng/mL WNT5A. Error bars represent the SD in five replicates. *$P < 0.05$, **$P < 0.01$ (Student's *t*-test). **(D)** Percentage of oocytes with different maturation morphologies in control and CVW-treated groups. **(E)** Combined effects of CVW and FLI on PB1 extrusion in our current IVM medium. Error bars represent the SD in four replicates. n.s., not significant. **$P < 0.01$ (Student's *t*-test).

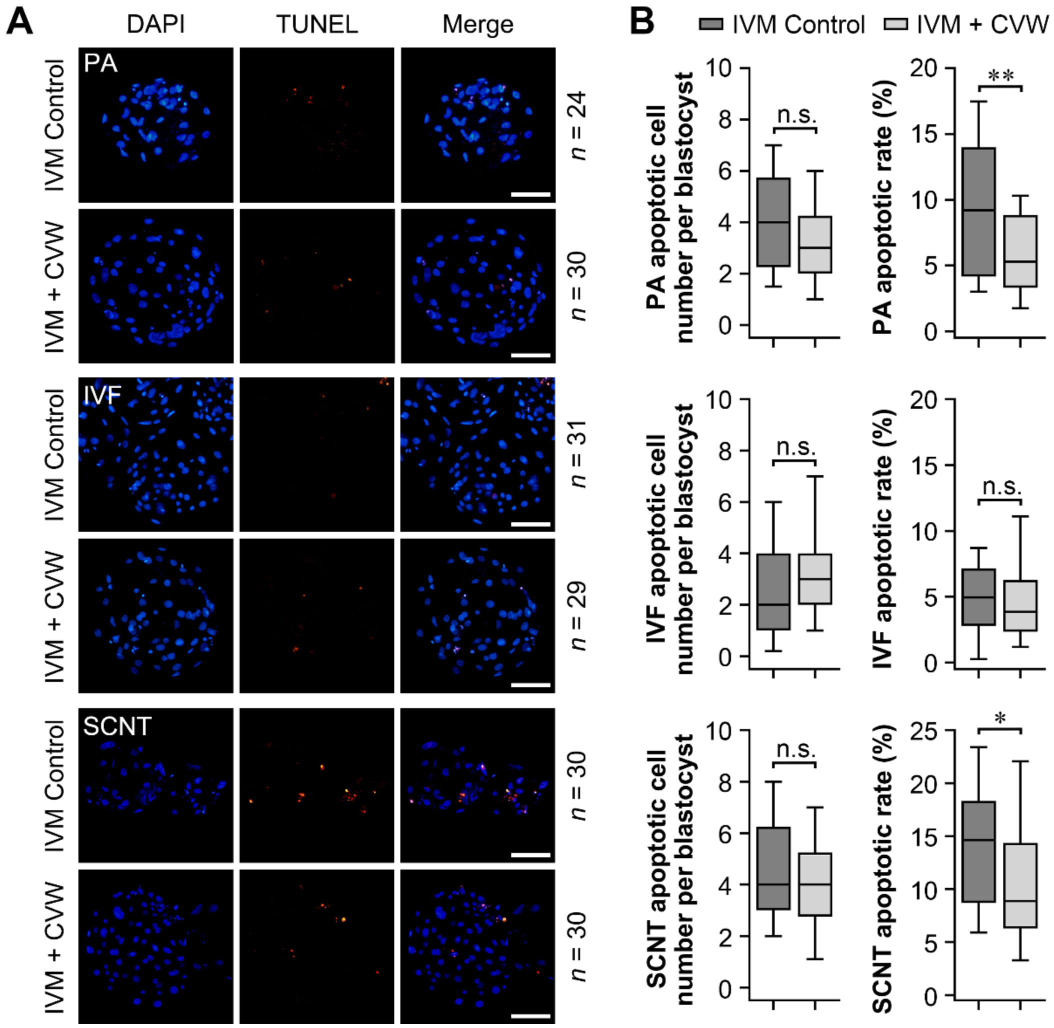

Supplementary Figure 3. Apoptosis in blastocysts derived from oocytes matured with or without CVW supplementation. **(A)** Immunostaining of TUNEL (red) and DAPI (blue) in PA, IVF and SCNT blastocysts derived from control and CVW-treated oocytes. Scale bars = 100 μm. **(B)** Box plots show the apoptotic cell numbers and apoptotic rates of PA, IVF and SCNT blastocysts derived from control and CVW-treated oocytes. All middle lines in box plots indicate the median, the edges indicate the 25th/75th percentiles and the whiskers indicate the 10th/90th percentiles. n.s., not significant. *$P < 0.05$, **$P < 0.01$ (Student's *t*-test).

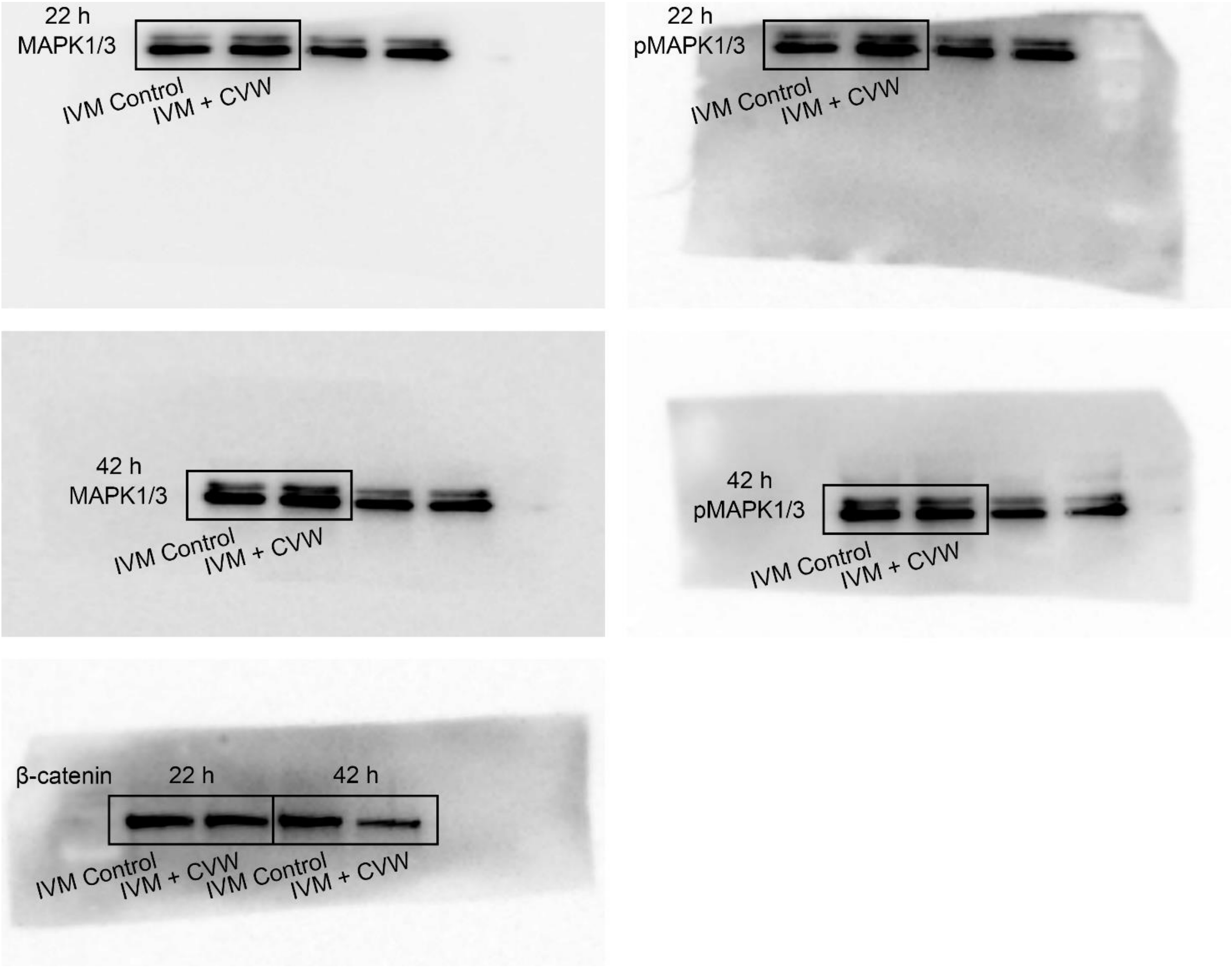

Supplementary Figure 4. The original polyvinylidene fluoride membranes of immunoblots. The immunoblots were visualized by using Pierce ECL Western Blotting Substrate, and captured on ImageQuant LAS 4000 imaging system.

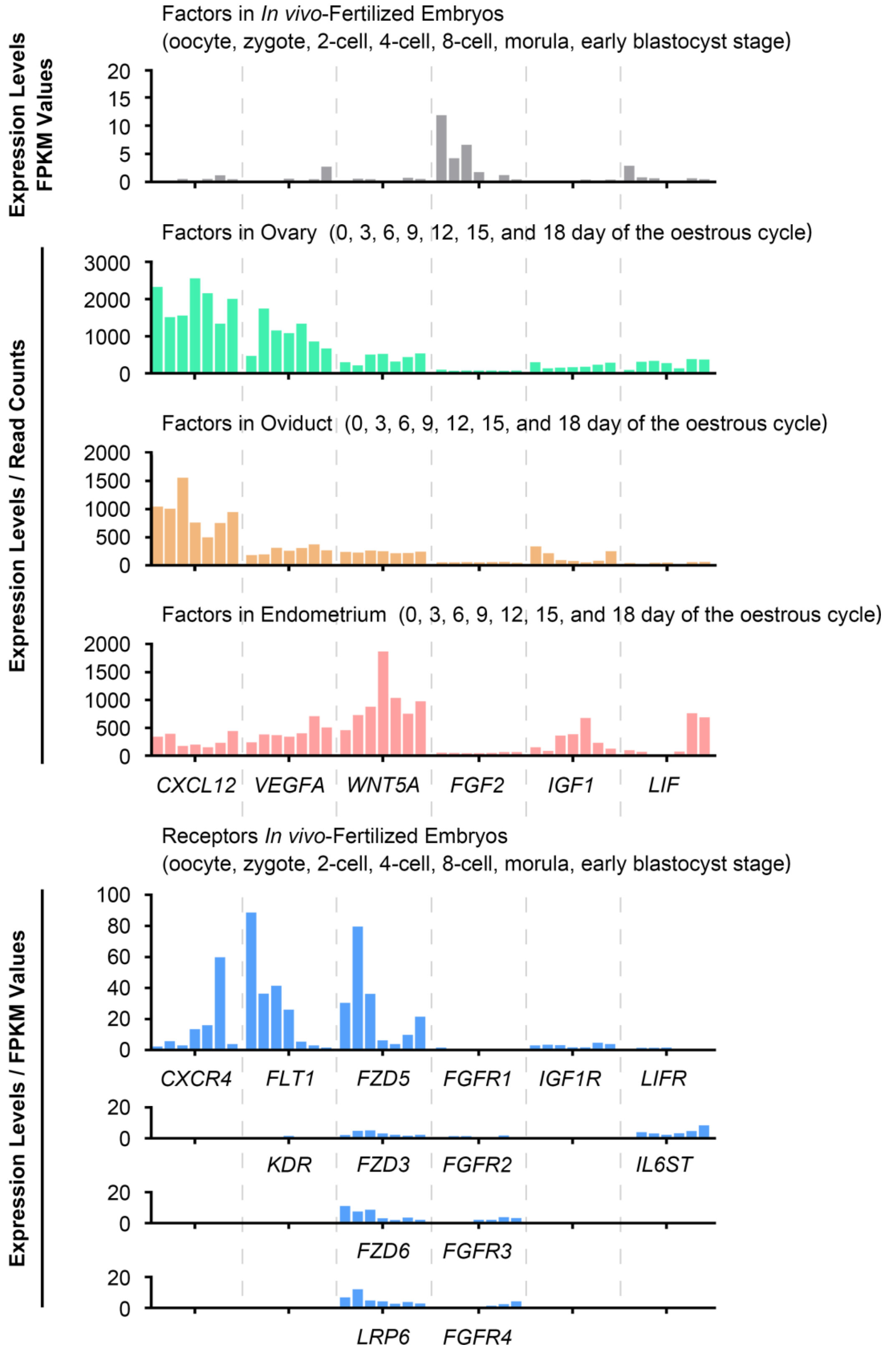

Supplementary Figure 5. Gene expression levels of CXCL12, VEGFA, WNT5A (CVW) and FGF2, LIF, IGF1 (FLI) in porcine *in vivo*-derived oocytes (gray), fertilized embryos (gray), ovary (green), oviduct (orange) and endometrium (pink) at different time points, and gene expression levels of their corresponding receptors in oocytes and fertilized embryos (blue) during the preimplantation stage.
